# Targeting Oxidative Stress as a Therapeutic Approach for Idiopathic Pulmonary Fibrosis

**DOI:** 10.3389/fphar.2021.794997

**Published:** 2022-01-21

**Authors:** Cristina Estornut, Javier Milara, María Amparo Bayarri, Nada Belhadj, Julio Cortijo

**Affiliations:** ^1^ Department of Pharmacology, Faculty of Medicine, University of Valencia, Valencia, Spain; ^2^ Pharmacy Unit, University General Hospital Consortium, Valencia, Spain; ^3^ CIBERES, Health Institute Carlos III, Valencia, Spain; ^4^ Research and Teaching Unit, University General Hospital Consortium, Valencia, Spain

**Keywords:** IPF—idiopathic pulmonary fibrosis, fibrosis, oxidative stress, ROS—reactive oxygen species, antioxidant therapy

## Abstract

Idiopathic pulmonary fibrosis (IPF) is a chronic interstitial lung disease characterized by an abnormal reepithelialisation, an excessive tissue remodelling and a progressive fibrosis within the alveolar wall that are not due to infection or cancer. Oxidative stress has been proposed as a key molecular process in pulmonary fibrosis development and different components of the redox system are altered in the cellular actors participating in lung fibrosis. To this respect, several activators of the antioxidant machinery and inhibitors of the oxidant species and pathways have been assayed in preclinical *in vitro* and *in vivo* models and in different clinical trials. This review discusses the role of oxidative stress in the development and progression of IPF and its underlying mechanisms as well as the evidence of oxidative stress in human IPF. Finally, we analyze the mechanism of action, the efficacy and the current status of different drugs developed to inhibit the oxidative stress as anti-fibrotic therapy in IPF.

## 1 Introduction

### 1.1 Idiopathic Pulmonary Fibrosis

Among all the idiopathic interstitial pneumonias, the most common form is idiopathic pulmonary fibrosis (IPF), whose incidence is estimated to be ranged between 2.8 and 9.3 per 100,000 people per year in North America and Europe (Barratt et al., 2018). IPF occurs primarily in older adults, mainly in men; it is characterised by chronic and progressive fibrosis related to a decrease in pulmonary function, progressive respiratory distress, and a remarkable poor prognosis. It is generally nonresponsive to traditional therapies such as anti-inflammatory and immunomodulatory treatment (Richeldi et al., 1941; Day, 2008; Barratt et al., 2018; Lederer and Martinez, 2018).

As the disease progresses, some pathological changes appear, including fibrotic lesions and accumulation of fibroblast in focal zones called “fibroblast foci,” which causes the characteristic “honeycomb” appearance. The etiologic stimulus that initiates the disease and the underlying mechanism of pulmonary fibrosis are still unknown or poorly understood. Current studies suggest that fibrosis may result from the presence of continuous stimuli (both endogenous and exogenous) or injury followed by an aberrant wound healing process and a dysregulated repair/remodelling of the lung (Kinnula et al., 2005; Kliment and Oury, 2010).

Lung fibrosis is associated with accumulation of fibroblasts, myofibroblast activation or differentiation, alveolar reepithelization, extracellular matrix (ECM) dysregulation, oxidative stress, and inflammation (Phan, 2002; Manni and Oury, 2014). Thus, fibrosis development is mediated by interactions between various cell types such as fibroblasts; myofibroblasts; epithelial cells, especially type I and II alveolar epithelial cells (AECs); mesothelial and mesenchymal cells, as well as immune system cells. Although most studies have focused on the fibrotic process and the proliferation of fibroblast and myofibroblast, several studies suggest that increased oxidative stress may play a major role in the development and progression of IPF (Bocchino et al., 2010; Liguori et al., 2018; Cameli et al., 2020).

This review addresses the importance of the balance of oxidants/antioxidants in the pathogenesis of pulmonary fibrosis as well as the evidence of oxidative stress in human IPF with emphasis on the pharmacological approach targeting oxidative stress.

### 1.2 Oxidative Stress in Idiopathic Pulmonary Fibrosis

Oxidative stress arises as a result of an imbalance between reactive oxygen species (ROS) and reactive nitrogen species (RNS) production and antioxidant defence that leads to cellular dysfunction and tissue damage (Hosseinzadeh et al., 2018b). ROS are highly reactive oxygen metabolites; some examples are superoxide anion (O_2_^•−^), hydroxyl radical (HO^•^), and hydrogen peroxide (H_2_O_2_). RNS are molecules derived from the reaction between nitric oxide (NO) and O_2_ and derivates, one example is peroxynitrite. RNS can generate oxidative stress but also the named nitrosative stress (Thomas et al., 2008; Otoupalova et al., 2020). Lungs, due to their anatomy, location, and function, are particularly susceptible to oxidative stress (Crapo, 2003; Kinnula et al., 2005; Hosseinzadeh et al., 2018b).

Exogenous oxidizing agents such as cigarette smoke, toxins, hyperoxia, asbestos fibres, drugs and radiation also induce the production of ROS/RNS. Exogenous or endogenous generated ROS/RNS may directly damage the alveolar epithelium, favouring fibrotic interstitial lung responses (Kinnula et al., 2005). In addition to directly damaging the lung epithelium, ROS/RNS may also favour the development of pulmonary fibrosis by altering the expression of mediators implicated in the pathogenesis of IPF, such as the pro-fibrotic growth factor, transforming growth factor *β* (TGF-β). This growth factor is known to be modulated by ROS, indeed, experimental studies have evidenced that ROS can increase the secretion of TGF-β from epithelial cells and directly activate it (Barcellos-Hoff and Dix, 1996; Bellocq et al., 1999; Pociask et al., 2004). In turn, TGF-β stimulates the proliferation of fibroblasts and its differentiation into myofibroblasts (Thannickal et al., 2003). Oxidants may also alter the nature of surrounding ECM (Larios et al., 2001). Both ROS and RNS play an important role in the regulation of ECM, degradation and turnover (Fu et al., 2003; Nelson and Melendez, 2004). In lungs, alveolar inflammatory cells including lymphocytes, macrophages and neutrophils also produce ROS/RNS. In IPF patients, in addition to these inflammatory cells, fibroblasts and myofibroblast produce high levels of ROS/RNS in response to cytokines and growth factors and are involved in the underlying mechanism of fibrosis development Table 1 (Bergeron et al., 2003; Waghray et al., 2005).

**TABLE 1:** Summary of the molecules of the redox system and the implicated process in the IPF participating cells.

Cell	Redox system molecule	Implicated process	Reference
(myo)fibroblasts	NOX4	↑αSMA ↑fibronectin ↑procollagen	Hecker et al. (2009); Griffith et al. (2009); Amara et al. (2010); Hecker et al. (2014); Jarman et al. (2014); Cameli et al. (2020)
		↑migration	
		↑profibrotic phenotype	
		↑senescence	
		↓apoptosis	
	iNOS (NO)	↑TGFβ ↑collagen ↑HSP47	Zeidler et al. (2004); Hsu et al. (2007); Kliment and Oury. (2010)
		↑ECM-degrading enzymes	
	mtROS	↑profibrotic genes	Jain et al. (2013)
	Nrf2	↓fibrotic progression	Artaud-Macari et al. (2013)
Epithelial cells	NOX4	↑EMT	Hecker et al. (2009); Cameli et al. (2020)
	mtROS	↑senescence ↑apoptosis	Yoon et al. (2005); Panduri et al. (2009); Taslidere et al. (2014); Rangarajan et al. (2017)
	H_2_O_2_	Mimics TGFβ	Rhyu et al. (2005)
	Catalase	↓H_2_O_2_ ↓fibroblast activation	Waghray et al. (2005)
	SOD	↓ECM oxidative degradation	Petersen et al. (2004)
Inflammatory cells	NOX2 and NOX4	↑ECM deposition	He et al. (2019)
VSMCs	NOX4	↑proliferation	Sturrock et al. (2006); Kato and Hecker. (2020)
		↑remodelling	
Endothelial cells	NOX 2 and NOX4	↑angiogenesis	Teng et al. (2012); Jarman et al. (2014)

αSMA, α-smooth muscle actin; ECM, extracellular matrix; EMT, epithelial-to-mesenchimal transition; HSP47, heat-shock protein 47; iNOS, inducible NOS; mtROS, mitochondrial ROS; NO, nitric oxide: NOS, nitric oxide synthase; NOX, NADPH, oxidases; Nrf2, nuclear factor erythroid 2-related factor 2; SOD, superoxide dismutase; TGFβ, transforming growth factor β.

The main producers of ROS/RNS include nicotinamide adenine dinucleotide phosphate oxidases (NADPH oxidases, NOXs), myeloperoxidase (MPO), xanthine oxidase, nitric oxide synthase (NOS), and the mitochondrial electron transport chain (Kinnula et al., 2005; Hosseinzadeh et al., 2018b). From the family members of the NOX, the isoforms Nox1, Nox2 and Nox4 have been found to be implicated in the pathogenesis of pulmonary fibrosis but not Nox3 (Masamune et al., 2008; Griffith et al., 2009; Amara et al., 2010). Nox4 is ubiquitously expressed in various cell types of lung tissues including macrophages and structural cells such as smooth muscle cells, endothelial cells, mesenchymal cells, and epithelial cells (Lee et al., 2010a; Harijith et al., 2017). In IPF patients, Nox4 is strongly expressed in fibroblast foci and increases the expression of α-smooth muscle actin (*α*-SMA), fibronectin and procollagen, which are the most characteristic profibrotic molecules (Griffith et al., 2009; Hecker et al., 2009; Amara et al., 2010). Furthermore, Nox4 plays a critical role in myofibroblast tissue repair functions and fibrogenesis (Hecker et al., 2009). It is well known that TGF-β induces Nox4-dependent ROS production, which, in turn, promotes fibroblast migration (Amara et al., 2010) and this ROS production is also involved in the acquisition of pro-fibrotic myofibroblast phenotypes, including differentiation, contraction, apoptotic resistance, and ECM deposition (Hecker et al., 2009; Cameli et al., 2020). Nox4 expression is also increased in IPF senescent fibroblasts/myofibroblasts (Jarman et al., 2014) and ROS generated by Nox4 promotes senescence and the apoptosis-resistant phenotype (Hecker et al., 2014). Hyperplastic type II AECs from IPF patients’ lungs highly express this NOX isoform (Amara et al., 2010) and Nox4-dependent ROS generation induces epithelial-to-mesenchymal transition (EMT) in alveolar epithelial cells (Hecker et al., 2009; Cameli et al., 2020). Other studies demonstrate that Nox4 is implicated in the profibrotic polarization of macrophages and the genetic removal of Nox4 in macrophages reduces ECM deposition protecting from induced pulmonary fibrosis (He et al., 2019). In addition, Nox4, along with Nox1, Nox2, is also expressed in vascular smooth muscle cells (VSMCs) (Huetsch et al., 2019). A study has reported that Nox4 is highly expressed in thickened pulmonary arteries in IPF patients (Pache et al., 2011). It has been proved that VSMCs are activated by TGF-β1 to induce Nox4, leading to increased VSMCs proliferation (Sturrock et al., 2006). Nox4 expression may be altered in VSMCs and is likely to mediate vascular remodelling that may generate pulmonary hypertension in lungs from IPF patients (Kato and Hecker, 2020). Vascular endothelial cells also express Nox4 (Bernard et al., 2014), ROS generated by Nox4 is implicated in the regulation of endothelial cell motility and angiogenesis. Furthermore, Nox4 expression is higher at sites of angiogenesis within fibrotic regions and adjacent to fibrotic foci (Jarman et al., 2014).

Whereas Nox4 is mainly expressed in fibroblasts and epithelial cells, Nox2, is primarily expressed by neutrophils and macrophages and, to a lesser extent, in structural cells (mesenchymal cells, smooth muscle cells, endothelial cells, and airway epithelial cells) (Harijith et al., 2017). In IPF patients, neutrophils show an increased expression of Nox2 subunits (Kato and Hecker, 2020) and genetic removal of some of these subunits partially protects from the development of fibrosis in lung fibrosis mice models (Manoury et al., 2005; Kato and Hecker, 2020). Nox2-mediated ROS in vascular endothelial cells is implicated in autophagy induction, which contributes to impaired angiogenesis (Teng et al., 2012).

In lungs, NOS is the major enzyme producer of NO. The NOS family includes three isoforms: endothelial (eNOS), neuronal (nNOS) and inducible (iNOS) (Ricciardolo et al., 2004). RNS are mostly generated by iNOS, which is expressed by a huge variety of cells from the respiratory system. In normal conditions, NO has biological roles such as the relaxation of smooth muscle cells in pulmonary and cardiovascular system, however high levels of NO^•^ and NO-derived species may interact with different molecules and modify their function (Ricciardolo et al., 2006). In IPF, the high levels of iNOS expression and nitrotyrosine production in fibroblasts, as well as epithelial cells and macrophages, leads to unusual nitrosative stress. This nitrostive stress modifies different proteins in the lungs facilitating fibrogenesis and progression of the disease (Saleh et al., 1997).

Different studies have demonstrated that NO signal pathway may enhance TGF-β expression in lung fibroblasts and increase the expression of collagen type I and heat-shock protein (HSP) 47 (Hsu et al., 2007; Kliment and Oury, 2010). Further studies have demonstrated NO also seems to promote the expression of ECM-degrading enzymes in fibroblasts (Zeidler et al., 2004; Kliment and Oury, 2010). Additionally, endothelial cells may be involved in lung fibrosis through the production of the free radical NO (Michiels, 2003).

As we have mentioned, mitochondria is a major source of ROS production due to the uncoupling of the electron transport chain (Osborn-Heaford et al., 2012) and this production increases with senescence and ageing (Lee et al., 2002; Kurundkar and Thannickal, 2016). ROS generated by mitochondria are released into the cytosol and have been proved to be crucial in mediating pulmonary fibrosis (Cheresh et al., 2013). The pro-fibrotic factor, TGF-β1, enhances mitochondrial ROS, which have been proven to induce the expression of pro-fibrotic genes during myofibroblast differentiation. Pulmonary fibroblasts from IPF patients show a higher generation of mitochondrial ROS and inhibition of the generation of mitochondrial ROS decreases pro-fibrotic gene expression (Jain et al., 2013). Mitochondrial ROS generation is also increased in bleomycin-induced pulmonary fibrosis (Kim et al., 2016). Additionally, the epithelial cell damage occurring in IPF is linked with increased mitochondrial ROS (Kuwano et al., 2003). In turn, TGF-β1 induces mitochondrial ROS generation through mitochondrial complex IV inhibition in lung epithelial cells (Yoon et al., 2005). Furthermore, upregulation of mitochondrial ROS by TGF-β1 induce senescence in lung epithelial cells (Yoon et al., 2005; Taslidere et al., 2014). It has been suggested that mitochondrial impairment represents a key process for epithelial cell apoptosis in lung fibrosis (Panduri et al., 2009; Rangarajan et al., 2017).

The lung counteracts the damage induced by ROS with a wide variety of antioxidant defences. This endogenous antioxidant system includes small-molecular-weight antioxidants [vitamin E, melatonin, glutathione (GSH), uric acid, etc], classic antioxidant enzymes [superoxide dismutases (SODs), catalase, and glutathione peroxidase (GPx)], other antioxidant enzymes [peroxiredoxins (PRXs), thioredoxins (TRXs)], phase II detoxifying enzymes [glutathione-S-transferase (GST) isozymes, NADP(H), quinone oxidoreductase (NQO1), etc], stress-response proteins [heme oxygenase (HO)-1, ferritin, etc.], mucins (MUC), and metal binding proteins (lactoferrin, transferrin, metallothionein, etc) (Walters et al., 2008). The nuclear factor erythroid 2-related factor 2 (Nrf2) induces the expression of most of the antioxidant and detoxifying enzymes, which makes this factor essential for activating the antioxidant defence system. Under normal conditions, Nrf2 is sequestered in the cytoplasm by binding to Kelch like-ECH-associated protein 1 (KEAP1). In response to stress signals, Nrf2 is released from KEAP1 and translocated into the nucleus, where induces the expression of hundreds of antioxidant genes. Additionally, Nrf2 regulates the expression of genes involved in inflammatory and fibrotic responses (Hybertson et al., 2011). Fibroblasts and myofibroblasts from IPF patients express lower levels of Nrf2 when compared to controls fibroblasts. Activators of Nrf2 inhibit TGF-β1-induced pro-fibrotic effects in IPF fibroblasts and attenuate pulmonary fibrosis in animal models (Artaud-Macari et al., 2013).

In lungs, these antioxidant enzymes are expressed by the bronchial and alveolar epithelial cells and macrophages (Kinnula et al., 2005) and different studies suggest that overexpression of some of these antioxidant enzymes may protect against pulmonary fibrosis (Kang et al., 2003; Gao et al., 2008).

AECs in the lungs produce catalase, which exerts its activity via reducing H_2_O_2_ and, therefore, inhibits H_2_O_2_-mediated fibroblast activation in IPF lungs (Waghray et al., 2005). As well as epithelial cells, inflammatory cells also express the antioxidant enzyme catalase. It has been reported that intratracheal administration of catalase in asbestos-treated mice prevents the development of pulmonary fibrosis by inhibiting the generation of H_2_O_2_ in inflammatory cells (Murthy et al., 2009).

All three isoforms of superoxide dismutase, including extracellular-SOD (EC-SOD), are highly expressed in the lungs. In addition, they play a critical role in induced pulmonary fibrosis models by preventing oxidative stress (Bowler et al., 2002; Rabbani et al., 2005). It has been evidenced that EC-SOD exerts anti-fibrotic effects in lungs through prevention of oxidative degradation of ECM, avoiding the destructive effects of ECM degradation products on pulmonary epithelial and mesenchymal cells (Petersen et al., 2004).

Reduced GSH, a low-molecular weight antioxidant, is synthesized by bronchial epithelial cells and alveolar macrophages. Different studies supported that TGF-β1 suppresses gene expression of glutamate cysteine ligases (GCL), an enzyme implicated in the biosynthesis of GSH, in alveolar epithelial cells (Arsalane et al., 1997; Jardine et al., 2002). Overexpression of the active form of TGF-β1 in mice induces lung fibrosis, and it is associated with downregulation of GCL gene expression, decreased GSH levels in BALF, and increased oxidative stress (Liu et al., 2012).

#### 1.2.1 Evidence of Oxidative Stress Biomarkers in IPF Patients

Given the accepted role of oxidative stress in IPF, it is essential to investigate the presence of oxidative stress biomarkers. These biomarkers could provide clues about the disease progression and prognosis as well as be useful in the clinical assessment of the patients. It was in 1987 when the presence of oxidative stress biomarkers was first described (Cantin et al., 1987) in IPF patients. Since then, several researchers have investigated oxidative stress indicators in this pathology, as is resumed in Table 2.

**TABLE 2:** Summary of the oxidative stress biomarkers analysed in different biological specimens of IPF patients.

Specimen	Comparison (n)	Biomarker	Lung function/severity disease correlation	Reference
ELF	IPF (15) vs. Ctrl (19)	↓tGSH	No correlation	Cantin et al. (1989)
		↔GSH/GSH + GSSG		
ELF	IPF (10) vs. Ctrl (19)	↓tGSH	N/A	Borok et al. (1991)
		↓GSH		
ELF	IPF (17) vs. Ctrl (14)	↓tGSH	No correlation	Meyer et al. (1994)
BALF		↔tGSH		
BALF	IPF (12) vs. Ctrl (31)	↓GSH	N/A	Rahman et al. (1999)
		↔GSSG		
		↓GSH/GSSG		
Sputum	IPF (16) vs. Ctrl (15)	↓tGSH	↑disease severity	Beeh et al. (2002)
Plasma			↓VC	
BALF	IPF (16) vs. Ctrl (20)	↔GSH	No correlation	Markart et al. (2009)
		↑GSSG		
Blood	IPF (22) vs. Ctrl (29)	↓tGSH	No correlation	Muramatsu et al. (2016)
		↓tGSH/GSSG		
		↑GSSG	↓FVC	
Blood	IPF (11) vs. Ctrl (9)	↓GSH	N/A	Veith et al. (2017)
		↔GSSG		
Serum	IPF (37) vs. Ctrl (6)	↑lipid peroxidation	↑disease severity	Jack et al. (1996)
			↓VC	
		↑TBARS	No correlation	
Plasma	IPF (12) vs. Ctrl (31)	↑MDA	N/A	Rahman et al. (1999)
BALF		↓TEAC		
BALF	IPF non-smokers (14) vs Ctrl non-smokers (9)	↑Carbonyl proteins	N/A	Lenz et al. (1996)
BALF	IPF (9) vs. Ctrl (5)	↑Carbonyl proteins	N/A	Lenz et al. (2004)
BALF	IPF (13) vs. Ctrl (5)	↑Carbonyl proteins	N/A	Rottoli et al. (2005)
BALF	IPF (15) vs. Ctrl (8)	↑Carbonyl proteins	N/A	Bargagli et al. (2007)
EBC	IPF (16) vs. Ctrl (15)	↑8-isoprostane	No correlation	Psathakis et al. (2006)
		↑H_2_O_2_	↓DLCO	
Plasma and urine	IPF (29) vs. Ctrl (6)	↑Pl-isoprostanes	N/A	Jackson et al. (2010)
		↔Ur- H_2_O_2_		
	IPF at rest (29) vs. IPF after physical exercise	↔Pl-isoprostanes		
		↓Pl-TAC		
		↑Ur-isoprostanes		
		↔Ur- H_2_O_2_		
EBC	IPF (20) vs. Ctrl (20)	↑8-isoprostane	No correlation	Chow et al. (2012)
		↔ NOx		
		↔ H_2_O_2_		
		↑3-NT	↓FVE1, ↓FVC, ↓VC, ↓TLC	
Serum and BALF	IPF (16) vs. Ctrl (17)	↑8-isoprostane	N/A	Malli et al. (2013)
EBC	IPF (6) vs. Ctrl (6)	↑8-isoPGF2α	N/A	Shimizu et al. (2014)
Plasma	IPF (21) vs. Ctrl (12)	↑hydroperoxides	↑dyspnea severity	Daniil et al. (2006)
			↓ FVC ↓DLCO	
Serum	IPF (43) vs. Ctrl (30)	↑hydroperoxides	↓FVC ↓DLCO	Matsuzawa et al. (2015)
			↑acute exacerbation	
BALF	IPF (16) vs. Ctrl (20)	↑uric acid	N/A	Markart et al. (2009)
		↑ascorbic acid		
		↑vitamin A		
		↑vitamin E		
Blood	IPF (11) vs. Ctrl (9)	↓uric acid (not sig)	N/A	Veith et al. (2017)
		↓ascorbic acid (not sig)		
		↓TEAC		
Lung tissue	IPF (10) vs. Ctrl (5)	↔ECSOD	N/A	Kinnula et al., 2006
	IPF fibrotic areas vs IPF normal areas	↓ECSOD		
Lung tissue	IPF (10) vs. Ctrl (310)	↔PrxII	N/A	Vuorinen et al. (2008)
Lung tissue	IPF (7) vs. Ctrl (7)	↔NRF2	N/A	Mazur et al. (2010)
		↑SRX1		
	IPF hyperplastic epithelium vs IPF normal epithelium	↑NRF2		
		↑KEAP1		

3-NT, 3-nitrotyrosine; BALF, bronchoalveolar lavage fluid; Ctrl, control; DLCO, diffusing capacity of the lungs for carbon monoxide; EBC, expired breath condensate; ECSOD, Extracellular Superoxide Dismutase; ELF, epithelial lining fluid; FEV, forced expiratory volume; FVC, forced vital capacity; GSH, glutathione; GSSG, oxidized glutathione; IPF, idiopathic pulmonary fibrosis; KEAP1, Kelch like-ECH-associated protein 1; MDA, malondialdehyde; N/A, not available; not sig, not significative; NOx, NADPH, oxidases; NRF2, nuclear factor erythroid 2-related factor 2; Pl, plasma; PrxII, peroxiredoxin II; SRX1, sulfiredoxin-1; TAC, total antioxidant capacity; TBARS, thiobarbituric acid reactive substances; TEAC, trolox equivalent antioxidant capacity; tGSH, total glutathione; Ur, urine.

GSH is one of the antioxidant small molecule par excellence and one of the most measured biomarkers. Levels of this antioxidant molecule and its oxidized form, GSSG, have been measured as indicators of oxidative stress in multiple diseases. Regarding IPF, most of the studies reported lower levels of total GHS (reduced (GSH) + oxidized (GSSG), tGSH) and reduced GSH in IPF patients than in controls (Cantin et al., 1989; Borok et al., 1991; Meyer et al., 1994; Rahman et al., 1999; Beeh et al., 2002; Muramatsu et al., 2016; Veith et al., 2017), just two studies found no differences in GSH or tGSH between IPF patients and controls (Meyer et al., 1994; Markart et al., 2009). GSSG levels are found to be similar between IPF patients and controls (Rahman et al., 1999; Veith et al., 2017) or higher in IPF (Markart et al., 2009; Muramatsu et al., 2016). Ratios between the different forms of GSH have also been measured and are shown in Table 2. Most of the studies found no correlation between GSH forms and lung function. However, Beeh et al. (2002) found an inverse relationship between GSH sputum levels and disease severity and a positive correlation between GSH and vital capacity (VC %), Muramatsu et al. (2016) also found an inverse correlation between the change in GSSG and the change in forced vital capacity (FVC).

Another marker for oxidative stress is lipid peroxidation, usually determined through levels of thiobarbituric acid reactive substances (TBARS), and among all the lipid oxidation products the most studied is the malondialdehyde (MDA). These biomarkers were found to be higher in IPF patients when compared to healthy controls (Jack et al., 1996; Rahman et al., 1999). Jack et al. (1996) also found a significant negative correlation between the changes in lipid peroxidation and the changes in VC (%). Isoprostanes are free radical–catalyzed prostaglandin isomers whose generation reflects lipid peroxidation *in vivo* and, thus, are biomarkers of oxidative stress (Lawson et al., 1999). Concentrations of isoprostanes, especially 8-isoprostane, have been found to be higher in IPF patients compared to controls (Psathakis et al., 2006; Jackson et al., 2010; Chow et al., 2012; Malli et al., 2013; Shimizu et al., 2014). In addition, carbonyl proteins serve as markers of oxidized proteins and concentrations have been found to be higher in IPF patients than in healthy in controls (Lenz et al., 1996; Lenz et al., 2004; Rottoli et al., 2005; Bargagli et al., 2007).

Some studies have also measured the levels of H_2_O_2_ and in some cases, concentrations of this marker are higher in IPF patients, and a negative correlation between H_2_O_2_ and diffusing capacity of the lungs for carbon monoxide (DLCO) was observed (Psathakis et al., 2006). Other studies, on the other hand, reported no differences between groups (Jackson et al., 2010; Chow et al., 2012).

Hydroperoxide measurements, though are less common, are also used as oxidative stress biomarkers. Concentrations of these biomarkers are significantly higher in IPF patients than in controls. It has also been found a significant positive correlation between concentrations of hydroperoxides and severity of dyspnea or acute exacerbation and a negative correlation between the concentrations of hydroperoxides, FVC, and DLCO (Daniil et al., 2006; Matsuzawa et al., 2015).

Small-molecular-weight antioxidant molecules play a significant role in lung antioxidant defences. These small molecules include GSH, as we have mentioned above, but also other molecules such as vitamins and acid uric, which are also used as oxidative stress biomarkers. A study performed by Markart et al. (2009) reported significantly higher levels of uric acid, ascorbic acid (vitamin C), retinol (vitamin A), and tocopherol (vitamin E) in IPF patients when compared to healthy controls. However, a more recent study has reported that concentrations of uric acid and vitamin C were slightly, but not significantly, lower in IPF patients than in controls (Veith et al., 2017).

The antioxidant capacity can be also measured as an oxidative stress biomarker and, in some studies, it has been found to be significantly lower in IPF patients (Rahman et al., 1999; Jackson et al., 2010; Veith et al., 2017). Another molecule useful in oxidative stress measurements is 3-nitrotyrosine (3-NT) and it has been found to be higher in IPF patients compared to controls. Additionally, it is reported that there is an inverse correlation between 3-NT concentrations and forced expiratory volume (FEV1) (%), FVC (%), VC (%), and total lung capacity (TLC, %) (Chow et al., 2012).

When the biological specimen used is lung tissue, the studied oxidative stress biomarker is usually the expression of proteins or transcription factors implicated in different antioxidant pathways, such as the antioxidant enzyme SOD or the transcription factor Nrf2. ECSOD expression was found to be significantly lower in fibrotic areas when compared to non-fibrotic areas (Kinnula et al., 2006). Furthermore, regarding the antioxidant enzyme peroxiredoxin (PRX) II, it was found that there was no major Prx II oxidation in IPF lungs compared with the normal lung (Vuorinen et al., 2008). On the other hand, Mazur et al. (2010) analysed the Nrf2 –sulfiredoxin-1 (SRX1) pathway. The authors found non-specific cell variability in the expression of the Nrf2 pathway in healthy and fibrotic lungs. By contrast, the expression of SRX1 was increased in IPF compared to controls. Furthermore, the morphometric evaluation revealed that Nrf2 and KEAP1 were significantly increased in the hyperplastic alveolar epithelium compared to the normal alveolar epithelium (Mazur et al., 2010).

A huge number of oxidative stress biomarkers have been described in patients with IPF. Here we have just highlighted the most commonly analysed. The evaluation of these biomarkers could help in the clinical assessment of patients with IPF.

## 2 Antioxidant Therapy in Idiopathic Pulmonary Fibrosis

Considering oxidative stress plays a central role in the development and progression of IPF, antioxidant therapies have been proposed for many years. There are a few publications that review the use of antioxidant molecules or NOX inhibitors, naturals and synthetics, as potential therapeutics for lung fibrosis (Kinnula et al., 2005; Day, 2008; Kato and Hecker, 2020; Wang et al., 2021). In this review, we have selected the most advanced studies that include *in vitro, in vivo* and human evidence that could be translated into future treatments of IPF Summarized in Table 3.

**TABLE 3:** Summary of potential therapeutic antioxidants for IPF reviewed in this study.

Drug	Class	Mechanism of action	Clinical trial identifier NCT	Reference
DPI	NOX inhibitor	Pan-NOXs inhibitor		O’Donnell et al. (1993); Kato and Hecker. (2020)
Vas2870	NOX inhibitor	Pan-NOXs inhibitor		Kato and Hecker, (2020)
GKT137831	NOX inhibitor	NOX4/NOX1 dual inhibitor	NCT03865927	Gaggini et al. (2011)
GKT136901	NOX inhibitor	NOX4/NOX1 dual inhibitors		Gaggini et al. (2011)
Apocynin	NOX inhibitor	NOX2 inhibitor		Heumüller et al. (2008); Augsburger et al. (2019)
		ROS scavenger		
Metformin	NOX inhibitor	NOX4 inhibitor		Sato et al. (2016); Rangarajan et al. (2018)
	Anti-diabetic			
NAC	Antioxidant enhancer	ROS scavenger	NCT00639496	Watchorn et al. (1998); Parmentier et al. (1999); Cuzzocrea et al. (2001); Sugiura et al. (2009); Ji et al. (2010)
		GSH precursor	NCT00650091	
		**---|** NFkB	UMIN000015508	
		↑ Nrf2	NCT02707640	
			NCT04300920	
			NCT03720483	
Quercetin	Antioxidant enhancer	ROS scavenger	NCT02874989	Tanigawa et al. (2007); Veith et al. (2017); Cazzola et al. (2018); Sellarés and Rojas. (2019)
	Senolytic	↑ Nrf2		
Salvianolic acid B	Antioxidant enhancer	ROS scavenger	NCT03274544	Xiao et al. (2020)
		↑ Nrf2		
		---| NOX2,4		
EGCG	Antioxidant enhancer	ROS scavenger	NCT03928847	Salah et al. (1995); Nanjo et al. (1999); Nagai et al. (2002a); Negishi et al. (2004); Zhang et al. (2007a); Sriram et al. (2009a)
		↑ Nrf2		
		↓Inflammatory mediators		
		↓Lipid peroxidation		
Tanshinone IIA and Sodium tanshinone IIA sulfonate	Antioxidant enhancer	ROS scavenger		Zhang and Wang. (2007); An et al. (2019)
		↑ Nrf2		
		↑GSH		
		---| NOX4		
Resveratrol	Antioxidant enhancer	ROS scavenger ↑ Nrf2		Zhu et al. (2017)
Sulforaphane	Antioxidant enhancer	↑ Nrf2		Elbarbry and Elrody. (2011); Kim and Park. (2016)
Melatonin	Antioxidant enhancer	ROS scavenger		Swiderska-Kołacz et al. (2006); Reiter et al. (2007); Santofimia-Castaño et al. (2015); Goc et al. (2017)
		↑ Nrf2		
		↓Inflammatory mediators		
Curcumin	Antioxidant enhancer	ROS scavenger		Lee et al. (2010b); Zhou et al. (2011); Lelli et al. (2017)
		↑ Nrf2		
		↑ Antioxidant molecules		
Pirfenidone	Antifibrotic	ROS scavenger	NCT00287716 NCT00287729	Giri et al. (1999); Misra and Rabideau. (2000); Nakazato et al. (2002); Oku et al. (2008); Ma et al. (2021)
	Antioxidant enhancer	↑ Nrf2	NCT01366209	
		↑ Antioxidant molecules		
		↓ Lipid peroxidation		
		↓Inflammatory mediators		
Thalidomide	Antiemetic	↑ Antioxidant molecules	NCT00162760	Amirshahrokhi. (2013); Dong et al. (2017)
	Antioxidant enhancer		NCT00600028	
Crocin	Antioxidant enhancer	↑ Nrf2		Zaghloul et al. (2019); Mehrabani et al. (2020)
		↑ Antioxidant molecules		
Isorhamnetin	Antioxidant enhancer	ROS scavenger		Chi et al. (2016); Zheng et al. (2019); Luo et al. (2019); Ren et al. (2021)
		↑ Nrf2		
Echinochrome A	Antioxidant enhancer	↑ Antioxidant molecules		Lebed’ko et al. (2015)
		↓Inflammatory mediators		
AEOL 10150	Catalytic antioxidant mimetics	Mimics SOD		Rabbani et al. (2007); Garofalo et al. (2014); MacVittie et al. (2017); Zhang et al. (2018b)
		Mimics CAT		
AEOL 10113	Catalytic antioxidant mimetics	Mimics SOD		Vujaskovic et al. (2002)
MnTBAP	Catalytic antioxidant mimetics	Mimics SOD		Oury et al. (2001); Venkatadri et al. (2017)

CAT, catalase; DPI, Diphenyleneiodonium; EGCG, Epigallocatechin gallate; NAC, n-acetyl cysteine; NFκβ, nuclear factor kappa beta; NOX, NADPH, oxidase; NRF2, nuclear factor erythroid 2-related factor 2; ROS, reactive oxygen species; SOD, superoxide dismutase.

### 2.1 NOX Inhibitors

#### 2.1.1 Diphenyleneiodonium

**FIGURE 1:**
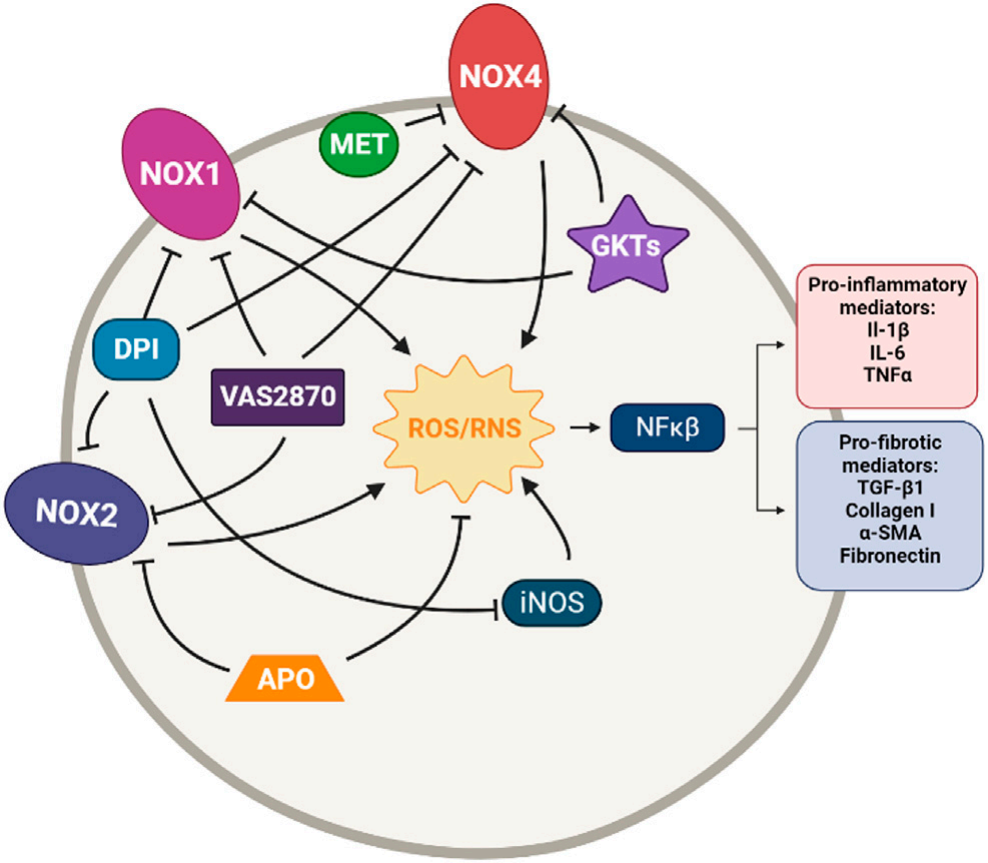
Simplified diagram of the principal molecular mechanisms of the NOX inhibitors Diphenyleneiodonium (DPI), VAS2870, GKT137831 and GKT136901 (GKTs), Apocynin (APO) and Metformin (MET). αSMA: alpha smooth muscle actin; IL-1β: interleuquina 1beta; IL-6: interleuquina 6; iNOS: inducible nitrogen oxide synthase; NFκβ: nuclear factor kappa beta; NOX1,2,4: NADPH oxidases; ROS/RNS: reactive oxygen species/reactive nitrogen species; TGF-β1: transforming growth factor beta 1; TNFα: tumoral necrosis factor alpha Created with Biorender.com.

Diphenyleneiodonium (DPI) is a potent inhibitor of NOX, which specifically and irreversibly binds to flavin, the membranous component of the NOX. It is the most commonly used and well-studied Nox inhibitor; however, its irreversible binding, lack of specific, poor solubility and toxicity *in vivo* do not make it the suitable candidate for a therapeutic option (O’Donnell et al., 1993; Kato and Hecker, 2020) (Figure 1).

Even so, DPI has been reported to inhibit collagen type I deposition and proliferation of pulmonary cells after stimulation with IPF sera (Fois et al., 2018). Another study has demonstrated that DPI decreases MPO activity, iNOS expression, intracellular ROS levels, the number of inflammatory cells, and cytokines TNF-α and IL-6 in lipopolysaccharide (LPS)-induced acute lung injury rats (Kim et al., 2019).

#### 2.1.2 VAS2870

Vas2870 was first described as a Nox2 inhibitor but later was described as a pan-NADPH oxidase inhibitor, with no selectivity for any NOX isoform (Wingler et al., 2012). It has been used in different cell models, but it has shown off-target effects due to its unspecific redox mode of action (Kato and Hecker, 2020). However, VAS2870 has been proved to reduce ROS generation restore epithelium barrier integrity and preserve cell viability in LPS-induced injury in alveolar epithelial cells (Li et al., 2020). It has also been reported to protect human pulmonary microvascular endothelial cells against LPS-induced inflammation through inhibiting the generation of ROS (Li et al., 2020); to inhibit phenotypic changes in fibrotic cells, including *α*-SMA and vimentin expression (Choi et al., 2016); and to suppress growth factor-mediated ROS liberation and migration in VSMC (ten Freyhaus et al., 2006) (Figure 1).

#### 2.1.3 GKT137831 and GKT136901

GKT137831 and GKT136901 were developed by Genkyotex (Geneva, Switzerland) through a high-throughput screening approach to discover small-molecule inhibitors targeting NOX enzymes (Laleu et al., 2010). Both small molecules are Nox4/Nox1 dual inhibitors. GKT137831 has been demonstrated to have strong antifibrotic activity at a low dose with much better efficacy than pirfenidone in curative model of bleomycin-induced pulmonary fibrosis in mice, as it is reported by the company (Gaggini et al., 2011). These Genkyotex compounds have also been reported to have protective effects in different pre-clinical *in vitro* and *in vivo* studies (Carnesecchi et al., 2011; Green et al., 2012; Jiang et al., 2012; Wan et al., 2016; Tanaka et al., 2017; Cui et al., 2018) (Figure 1).

In 2010, GKT137831 was granted orphan drug status for the treatment of IPF by the European Commission and is currently in clinical trials for IPF. A phase 2 clinical trial of GKT137831 [GKT137831 in IPF Patients With Idiopathic Pulmonary Fibrosis (GKT137831)] has recently started and it is a placebo-controlled, multicentre, randomized trial to test GKT137831 in ambulatory patients with IPF. The primary outcome is the reduction of the circulating concentrations of o,o’-dityrosine, an oxidative stress biomarker. Changes in concentrations of the collagen degradation and FVC are some of the secondary outcomes (ClinicalTrials.gov Identifier: NCT03865927).

#### 2.1.4 Other NOX Inhibitors

Some other molecules are also claimed to be NOX inhibitors. It is the case of apocynin, a natural organic compound obtained from plants, that was found to have therapeutic effects in animal models of various diseases (Virdis et al., 2016). In particular, it shows a protective and therapeutic effect on bleomycin-induced lung fibrosis in rats (Kilic et al., 2015). However, although this compound is usually defined as a Nox2 inhibitor, several studies have reported that apocynin would have intrinsic antioxidant properties rather than be a Nox inhibitor (Heumüller et al., 2008; Augsburger et al., 2019). On the other hand, we have metformin, an anti-diabetic drug not known primarily for its antioxidant potential, but a recent study has demonstrated that treatment with metformin inhibits TGF-β1–induced Nox4 expression, ROS generation and myofibroblast differentiation in lung fibroblasts *in vitro* and also attenuates bleomycin-induced lung fibrosis (Sato et al., 2016; Rangarajan et al., 2018). Despite the lack of clinical trials regarding the efficacy of metformin in the treatment of IPF, there is a retrospective study in humans treated with pirfenidone along with metformin; however, its results are not particularly promising (Spagnolo et al., 2018). Only future trials could provide more clues about this (Figure 1).

On the other hand, numerous groups are currently working on the finding or development of new NOX inhibitors that may become a therapeutic option in treating IPF.

### 2.2 Antioxidant Enhancers and Reactive Oxygen Species Scavenger

#### 2.2.1 N-Acetyl Cysteine

**FIGURE 2:**
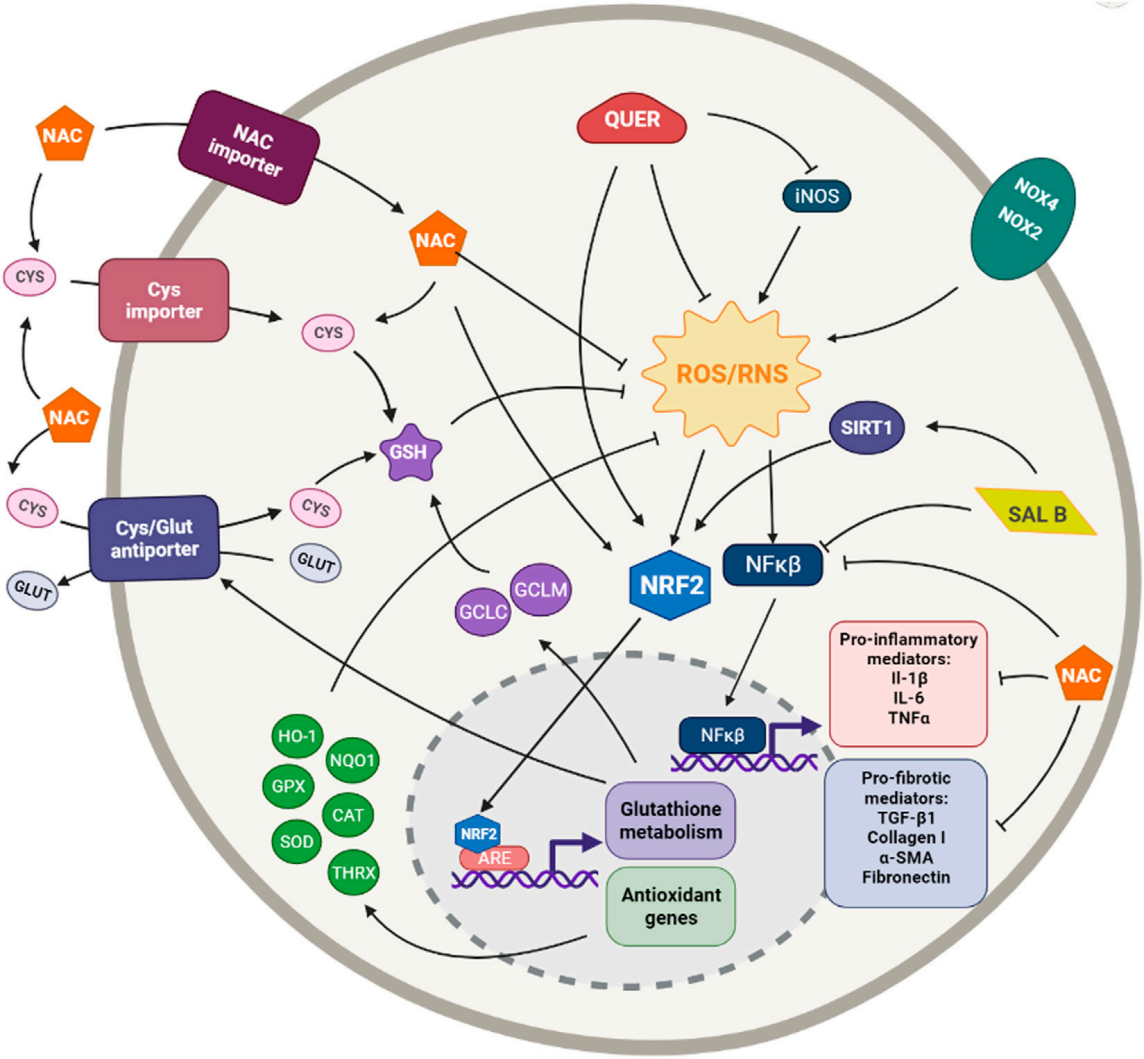
Simplified diagram of the principal molecular mechanisms of the antioxidant enhancers n-acetyl cysteine (NAC), Quercetin (QUER) and Salvianolic acid B (SAL B). αSMA: alpha smooth muscle actin; ARE: antioxidant responsive element; CAT: catalase; CYS: cysteine; GCLC: glutamate cysteine ligase catalytic subunit; GCLM: glutamate cysteine ligase modifier subunit; GLUT: glutamate; GPX: glutathione peroxidase; GSH: glutathione; HO-1: heme oxygenase 1; IL-1β: interleuquina 1beta; IL-6: interleuquina 6; iNOS: inducible nitrogen oxide synthase; NFκβ: nuclear factor kappa beta; NOX1,2,4: NADPH oxidases; NQO1: NAD(P)H:quinone oxidoreductase 1; NRF2: nuclear factor erythroid 2-related factor 2; ROS/RNS: reactive oxygen species/reactive nitrogen species; TGF-β1: transforming growth factor beta 1; TNFα: tumoral necrosis factor alpha; SIRT: sirtuine 1; SOD: supeoxide dismutase Created with Biorender.com.

N-acetyl cysteine (NAC) is an l-cysteine derived aminoacid with powerful reductive capacity. This aminothiol is not only a GSH precursor but also presents a direct ROS-scavenging capacity and may induce Nrf2 expression (Cuzzocrea et al., 2001; Sugiura et al., 2009; Ji et al., 2010). These properties have made NAC a broadly used potent antioxidant (Figure 2).

##### 2.2.1.1 *In vitro* Cellular Studies

Several *in vitro* assays have demonstrated that NAC prevents GSH depletion in various cell types such as macrophages, epithelial cells, and fibroblasts (Watchorn et al., 1998; Parmentier et al., 1999; Liu et al., 2004; Felton et al., 2009). NAC has also been reported to inhibit collagen production and EMT in TGF-β1-stimulated murine embryo fibroblasts and rat alveolar epithelial cells, respectively (Liu et al., 2004; Felton et al., 2009). A further study found that NAC significantly diminishes TGF-β1-induced fibronectin and VEGF production as well as *α*-SMA expression in human lung fibroblasts (Sugiura et al., 2009). These studies implied that NAC may affect the TGF-β1-induced tissue remodelling or fibrotic process *in vitro*.

Numerous studies have demonstrated that NAC inhibits the production of different inflammatory mediators such as tumour necrosis factor alpha (TFNα), interleukin-8 (IL-8) and matrix metalloproteinase-9 (MMP-9) in epithelial cells, macrophages, and lymphocytes from patients with IPF (Watchorn et al., 1998; Parmentier et al., 1999; Cu et al., 2009; Radomska-Leśniewska et al., 2010).

##### 2.2.1.2 *In vivo* Animal Studies

The first study that demonstrated the antifibrotic effect of NAC was performed by Shahzeidi et al. (1991). They showed that NAC inhibits collagen accumulation in lungs from rats with bleomycin-induced lung fibrosis (Shahzeidi et al., 1991). Since then, several studies have reported that NAC inhibits several profibrotic mechanisms in bleomycin-induced fibrosis murine models (Myllärniemi and Kaarteenaho, 2015). For instance, aerosolized administration of NAC attenuated bleomycin-induced lung fibrosis in mice *via* the decrease of the amounts of hydroxyproline, fibrosis and several cytokines’ levels (Hagiwara et al., 2000). Further studies performed by our group showed that treatment with NAC, in bleomycin-exposed rats, decreased the augmented collagen deposition and the inflammatory cells numbers. It also increased GSH levels and decreased MUC5a expression, fibrotic areas, TNF-α levels and MPO activity (Cortijo et al., 2001; Mata et al., 2003; Serrano-Mollar et al., 2003).

More recent animal studies on NAC using lung fibrosis murine models have been performed. In 2012, a study revealed that NAC treatment reversed lysyl oxidase activity to normal levels and increased GSH levels in the lung of bleomycin-induced rats, inhibiting TGF-β1 and α-SMA expression, thus attenuating pulmonary fibrosis (Li et al., 2012). A posterior study demonstrated that administration of NAC-pre-treated human mesenchymal stem cells to nude mice with bleomycin-induced lung injury decreased the pathological grade of lung inflammation and fibrosis, hydroxyproline content and numbers of neutrophils and inflammatory cytokines in BALF and apoptotic cells (Wang et al., 2013). The therapeutic potential of NAC in pulmonary fibrosis was also studied in rats exposed to silica particles. It was demonstrated that NAC treated silica-exposed rats showed significantly lower fibrosis scores, as well as lower levels of hydroxyproline amounts and MDA. NAC also attenuated silica-induced increments in TNF-α, IL-8, high-sensitivity C-reactive protein and ROS content (Zhang et al., 2013; Zhang et al., 2014a).

##### 2.2.1.3 Human Clinical Trials

In contrast to most cases of drug development, animal and human trials with NAC were conducted even earlier than *in vitro* ones. *In vitro* assays have shown, indeed, that NAC reduces fibrotic and remodelling processes in fibrosis models. Nevertheless, previously, *in vivo* animal trials had already suggested that NAC could be a good candidate for clinical trials. All clinical trials regarding NAC are summarized in Table 4.

**TABLE 4:** Summary of clinical trials of antioxidant therapies of IPF.

Drugs	Identifier (acronym)	Study desing, sample size	Primary endpoint	Secondary endpoints	Outcome	Observations
GKT137831 (400 mg b.i.d) Vs.Placebo	NCT03865927	Phase 2 Randomized Double-Blind	Changes in concentrations of circulating o,o'-dityrosine at 24 weeks	Changes in concentrations of the collagen degradation product	Recruiting	
		Parallel Assignment		FVC, 6MWD at 24 weeks		
		Placebo-Controlled *n* = 60				
NAC (oral 600 mg t.i.d) + prednisone + azathioprine Vs. Placebo + prednisone + azathioprine	NCT00639496 (IFIGENIA)	Phase 3 Randomized Double-Blind	Changes in VC and DLCO at 6 and 12 months	CRP-score at 6 and 12 months	Completed. Three-drug therapy preserved VC and DLCO	
		Parallel Assignment				
		Placebo-Controlled *n* = 184				
NAC (oral 600 mg t.i.d) + prednisolone + azathioprine Vs. NAC (oral 600 mg t.i.d) + placebo Vs. placebo	NCT00650091 (PANTHER)	Phase 3 Randomized Double-Blind	Changes in FVC at 60 weeks	The time-to-death or a 10% decline in FVC, acute exacerbations, respiratory infections at 60 weeks	Completed. NAC offered no significant benefit	Three-drug regimen was stopped due to safety concerns
		Parallel Assignment				
		Placebo-Controlled *n* = 264				
NAC (inhaled 352.4 mg b.i.d) + pirfenidone Vs. placebo + pirfenidone	UMIN000015508	Phase 3 Randomized Open-label	Changes in FVC at 48 weeks	Changes in 6MWD, VC, TLC, DLCO at 48 weeks	Completed. Combination therapy did not bring any clinical benefit	
		Parallel Assignment				
		Placebo-Controlled *n* = 150				
NAC (oral 600 mg t.i.d) + pirfenidone Vs. Placebo + pirfenidone	NCT02707640 (PANORAMA)	Phase 2 Randomized Double-Blind	Assessment of adverse events at 24 weeks	Changes in FVC, DLCO, 6MWD at 24 weeks	Completed. NAC does not alter tolerability profile of pirfenidone and is unlikely to be beneficial	
		Parallel Assignment				
		Placebo-Controlled *n* = 123				
NAC (oral 600 mg t.i.d) Vs. Placebo	NCT04300920 (PRECISIONS)	Phase 3 Randomized Double-Blind	10% relative decline FVC, first respiratory hospitalization, lung transplant or death from any cause at 24 months	Time to first all-cause hospitalization, annualized rate of respiratory hospitalizations, changes in DLCO at 24 months	Recruiting	
		Parallel Assignment				
		Placebo-Controlled *n* = 200				
NAC (inhaled) Vs. placebo	NCT03720483	Phase 1/2 Randomized Open-label	Changes in FVC at week 10 and 18	Changes in DLCO at week 10 and 18	Withdrawn	The study encountered challenges during startup due to the COVID-19 epidemic and was withdrawn
		Crossover Assignment, *n* = 0				
Dasatinib (100 mg/d) + Quercetin (1250 mg/d) Vs. Placebo	NCT02874989	Phase 1 Randomized Open-label	Retention rates and completion rates for planned clinical assessments (e.g., percentage of pro-inflammatory expressing cells, blood pressure, weight, heart rate) at 4 weeks	Safety and change in functional and reported health measures	Completed. Senolytics improved 6MWD	
		Parallel Assignment, Placebo-Controlled *n* = 26				
PROLUNG (contains Salvianolic acid B)	NCT03720483	Open-label Single Group Assignment *n* = 6	Change in FVC at 6 months	Changes in SGRQ score, SF-36 score, WHOQOL-BREF score and in adverse events over 6 months	Terminated	Difficulty in recruiting eligible patients
EGCG (600 mg daily)	NCT03928847	Early Phase I Open-label Single Group Assignment *n* = 35	LOXL2 activity and TGFbeta1 signaling biomarkers such as Snail1 and pSmad3 at 2 weeks	Maximum plasma concentration of EGCG 0, 2, 4, 12 h post dose and adverse events at 2 weeks	Recruiting	
Pirfenidone (1197 or 2403 mg in divided doses t.i.d) Vs. placebo	NCT00287716 (CAPACITY: study 004	Phase 3 Randomized Double-Blind	Changes in FVC at 72 weeks	PFS, changes in 6MWD, SpO2, DLCO, dyspnea score and worsening of IPF at 72 weeks	Completed. Improvement in lung function, in PFS, and in the associated death	
		Parallel Assignment				
		Placebo-Controlled *n* = 435				
Pirfenidone (2403 mg in divided doses t.i.d) Vs. placebo	NCT00287729 (CAPACITY: study 006)	Phase 3 Randomized Double-Blind	Changes in FVC at 72 weeks	PFS, changes in 6MWD, SpO2, DLCO, dyspnea score and worsening of IPF at 72 weeks	Completed. Improvement in lung function, in PFS, and in the associated death	
		Parallel Assignment				
		Placebo-Controlled *n* = 344				
Thalidomide (400 mg daily)	NCT00162760	Phase 2 Non-Randomized Open-label Single Group Assignment *n* = 19	Safety, feasibility and efficacy of thalidomide administered daily for 1 year	changes in pulmonary function tests, radiographs, dyspnea scales and quality of life measures	Completed. No results available	
Thalidomide (50–100 mg daily) Vs. Placebo	NCT00600028	Phase 3 Randomized Double-Blind	Suppression of cough measured by the CQLQ at 6 months	Suppression of cough measured by the VAS at 6 months	Completed. Thalidomide improved cough and respiratory quality of life	
		Crossover Assignment				
		Placebo-Controlled *n* = 344				

6MWD, 6-min walk distance; b.i.d, bis in die, twice a day; CQLQ, Cough Quality of Life Questionnaire; CRP -score, clinical, radiologic and physiologic score; DLCO, diffusion capacity for CO; EGCG, Epigallocatechin-3-gallate; FVC, forced vital capacity; NAC, N-acetylcysteine; PFS, progression-free survival; SF-36, 36-Item Short Form Survey; SGRQ, St. George’s Respiratory Questionnaire; SpO2, oxygen saturation by pulse oximetry; t.i.d, ter in die, three times a day; TLC, total lung capacity; VAS, visual analog scale of cough; VC, vital capacity; WHOQOL-BREF, world Health Organization Quality of Life abbreviated version.

In the 1990s, several open-label studies were conducted to analyse the efficacy of the short-term treatment of NAC in patients with various types of pulmonary fibrosis. These studies reported that total GSH levels increased and pulmonary function tests significantly improved after therapy with NAC (Meyer et al., 1994; Meyer et al., 1995; Behr et al., 1997).

The first clinical trial that assesses the effectiveness of NAC in IPF therapy was the IFIGENIA, which purpose was to determine whether NAC added to prednisone and azathioprine was more effective than the standard therapy with prednisone plus azathioprine. This study showed the three-drug therapy preserved VC and DLCO in IPF patients better than standard therapy (Demedts et al., 2005) (ClinicalTrials.gov Identifier: NCT00639496).

In the IFIGENIA trial, all patients received the combination of the three drugs but none, NAC alone. Thus, in order to study the effectiveness of NAC monotherapy for the treatment of IPF, it was conducted the PANTHER trial. Initially, subjects who have IPF were randomly assigned to receive: 1) three-drug regimen (NAC, prednisolone, and azathioprine), 2) NAC and placebo, or 3) placebo. After performing a midpoint analysis of the study, the three-drug regimen was stopped due to safety concerns. For this reason, the entire study was interrupted for 3 months but later continued. The primary outcome was the change in FVC over 60 weeks. Results showed that acetylcysteine offered no significant benefit concerning the preservation of FVC in patients with IPF when compared with placebo (Izumi et al., 2012; Martinez et al., 2014) (ClinicalTrials.gov number, NCT00650091).

Following this line, a phase III clinical trial evaluating the efficacy and safety of combined therapy with pirfenidone and inhaled NAC for IPF was conducted in Japan. The primary outcome was a change in FVC. Data showed there was no difference between the two groups in the change in FVC. Therefore, combination therapy did not bring any clinical benefit (Sakamoto et al., 2021) (University Hospital Medical Information Network registration number UMIN000015508). In addition to this study, a similar phase II trial was conducted, the PANORAMA study. The aim was to assess the safety and tolerability of NAC in IPF patients receiving background pirfenidone therapy. Again, findings suggested that the addition of NAC to pirfenidone does not substantially alter the tolerability profile of pirfenidone and is unlikely to be beneficial in IPF (Behr et al., 2016) (ClinicalTrials.gov Identifier: NCT02707640).

In 2015, a post hoc exploratory analysis of subjects enrolled in the PANTHER-IPF clinical trial was conducted to determine whether specific polymorphisms in toll-interacting protein (*TOLLIP*) and *MUC5B* genes modified the efficacy of NAC. These genes have been associated with IPF susceptibility and survival. TOLLIP encodes toll-interacting protein (TOLLIP), which is inhibitory of toll-like receptors, which are, in turn, responsible for the activation of inflammatory, oxidative and immune response pathways. This post hoc study showed that NAC might improve prognosis in genetically predisposed individuals, specifically, those carrying an rs3750920 (TOLLIP) TT genotype (Oldham et al., 2015). In order to assess this hypothesis, it has been proposed a genotype-stratified clinical trial: the PRECISIONS trial. The purpose of this study is to compare the effect of NAC plus standard care in patients diagnosed with IPF who have the TOLLIP rs3750920 TT genotype and it will compare the time to a composite endpoint of relative decline in lung function. Recruitment is ongoing and the study is estimated to finish in 2025. Thus, no results are published yet (ClinicalTrials.gov Identifier: NCT04300920).

Furthermore, a further phase I/II open label pilot study has been recently proposed to investigate the safety and tolerability of inhaled NAC in patients with IPF. It is titled “Pilot Study to Evaluate Inhaled N-Acetylcysteine in Pulmonary Fibrosis.” The primary outcomes are changes in pulmonary function: FVC and DLCO. This study faced different challenges during enrolment due to the COVID-19 epidemic and it is withdrawn at this moment. It is estimated to start at the beginning of 2022 and finish at the end of 2023 (ClinicalTrials.gov Identifier: NCT03720483).

#### 2.2.2 Quercetin

The antioxidant quercetin (3,3′,4′,5,7-pentahydroxyflavone) is a polyphenolic plant flavonoid ubiquitously present in vegetables and fruit as well as tea and red wine (Formica and Regelson, 1995; D’Andrea, 2015). It is a potent direct ROS scavenger but, also, indirectly, exerts its antioxidant function *via* activating the Nrf2 pathway and inducing Nrf2-regulated genes, such as NQO1, HO-1, GPX1, etc. (Tanigawa et al., 2007; Veith et al., 2017). However, quercetin has not only strong antioxidant but also anti-inflammatory capacities (Boots et al., 2008; Impellizzeri et al., 2015) (Figure 2).

Quercetin is also known as a senolytic drug, which induces selective elimination of senescent cells. It is usually used in combination with dasatinib, a tyrosine kinase inhibitor used in the treatment of some cancers (Cazzola et al., 2018; Sellarés and Rojas, 2019).

##### 2.2.2.1 *In vitro* Cellular Studies

Different studies have reported that quercetin induces expression of HO-1 in macrophages, preventing H_2_O_2_-induced apoptosis (Chow et al., 2005) and in mouse fibroblasts and normal human lung fibroblasts suppressing TGF-β-induced collagen production (Nakamura et al., 2011). Quercetin was also reported to inhibit the liberation of inflammatory cytokines such as TNF-α, IL8 and IL6 in macrophages (Manjeet and Ghosh, 1999) and alveolar epithelial cells (Geraets et al., 2007; Gauliard et al., 2008).

Furthermore, several investigations have demonstrated that quercetin has antifibrotic properties and inhibit skin, liver, or kidney fibrosis (Lee et al., 2003; Phan et al., 2003; Ren et al., 2016). In lung cells, quercetin has been reported to inhibit proliferation and expression of TBFβ1 in human embryonic lung fibroblasts activated by silicotic alveolar macrophages (Peng et al., 2013). Quercetin also suppresses bleomycin-induced EMT and intracellular level of ROS in alveolar type II-like cells (Takano et al., 2020). It has also been that this flavonoid ameliorates pulmonary fibrosis in TGF-β-treated human embryonic lung fibroblast (Zhang et al., 2018c).

Finally, quercetin along with dasatinib have been proved to be a powerful senolytic cocktail. Studies *in vitro* and *ex vivo* using primary fibrotic mouse alveolar epithelial type II and primary human fibroblasts demonstrated that treatment with this senolytic combination attenuates fibrotic mediator expression, such as senescence-associated secretory phenotype factor and extracellular matrix markers (Lehmann et al., 2017; Schafer et al., 2017).

##### 2.2.2.2 *In vivo* Animal Studies

Several studies have highlighted the protective effect of quercetin in various pulmonary fibrosis models, exercising anti-inflammatory and antifibrotic effects. In 2008, a study demonstrated that quercetin, although not influencing collagen deposition, attenuates the pulmonary oxidative stress and inflammatory in bleomycin-induced lung fibrosis hamster model (Martinez et al., 2008). Liposomal quercetin was demonstrated to attenuate bleomycin-induced pulmonary fibrosis *in vivo* by the suppression of inflammatory cytokines (TNF-α, IL-1β, and IL-6) and the diminish of total cells and macrophage counts in BALF. Moreover, treatment with liposomal quercetin produced a significant reduction of hydroxyproline content and apparently lessened areas of lung fibrosis and collagen deposition (Baowen et al., 2010). Furthermore, quercetin treatment was shown to reduce the expression of collagen, fibronectin, and MMP-7, decrease the level of inflammatory cytokines such as TNF-α and enhance Nrf2-induced pulmonary antioxidant defences (Verma et al., 2013; Boots et al., 2020). Thus, this demonstrates quercetin exerts anti-fibrogenic and anti-inflammatory effects, possibly *via* modulation of the redox balance by inducing Nrf2.

The effect of quercetin was also studied on pulmonary fibrosis induced by silica particles in rat models. Quercetin was demonstrated to reduce hydroxyproline content, and increase catalase and GPx activity (Liu et al., 2014a). Quercetin and dihydroquercetin also showed a protective effect against inflammatory processes associated with pulmonary fibrosis in bleomycin mice models. It was reported inhibition of oedema formation and body weight loss, as well as amelioration of polymorphonuclear infiltration into the lung tissue and reduction of the number of inflammatory cells in BALF. Moreover, these polyphenols suppressed iNOS, preventing oxidative and nitrosative lung injury (Impellizzeri et al., 2015). Moreover, these polyphenols suppressed iNOS, preventing oxidative and nitrosative lung injury (Impellizzeri et al., 2015).

The effects of quercetin have also been studied in combination with other compounds. Schafer et al. (2017) demonstrated that the combination of quercetin with dasatinib, the senolytic cocktail, attenuates bleomycin-mediated lung injury in mice. Quercetin has also been combined with gallic acid, another potent natural antioxidant, to investigate their protective effect against bleomycin-induced pulmonary fibrosis in rats. The combination treatment demonstrated a remarkable decrease in lung hydroxyproline and TNF-α level and an increase in catalase activity. The combination treatment also significantly enhanced lung SOD activity and GSH level and decreased NO and IL-6 levels (Mehrzadi et al., 2020).

##### 2.2.2.3 Human Clinical Trials

As mentioned in the previous paragraphs, quercetin is able to reduce oxidative stress and fibrotic processes such as EMT in different IPF effector cells. In addition, several *in vivo* studies have reported that quercetin has antifibrotic and anti-inflammatory effects through modulation of the redox balance probably by activation of Nrf2, among others. However, there are no human clinical trials testing quercetin treatment in IPF patients. On the other hand, the beneficial effect of quercetin supplementation on markers of oxidative stress and inflammation in other interstitial lung diseases, such as sarcoidosis, has been studied in different clinical trials (Boots et al., 2009; Boots et al., 2011) (ClinicalTrials.gov Identifier: NCT00402623 and NCT00512967).

Nevertheless, since *in vitro* and *in vivo* studies have demonstrated that the senolytic cocktail of quercetin and dasitinib is effective in IPF models, between 2016 and 2018, it was performed the first-in-human, small scale, pilot clinical trial to assess the feasibility, acceptability, best methods, and measurement characteristics of potential study outcomes for the senolytic drug combination dasatinib and quercetin in stable IPF patients. It was titled “Targeting Pro-Inflammatory Cells in Idiopathic Pulmonary Fibrosis: a Human Trial (IPF)” The primary endpoints were retention rates and completion rates for planned clinical assessments. Secondary endpoints were safety and change in functional and reported health measures. This first-in-humans open-label pilot study provided initial evidence that senolytics may improve the 6-min walk distance in IPF (Justice et al., 2019) (ClinicalTrials.gov identifier: NCT02874989) Summarized in Table 4.

#### 2.2.3 Salvianolic Acid B

Salvianolic acid B (Sal B) is one most active phenolic acids extracted from *Salvia miltiorrhiza,* Danshen. It shows strong antioxidant, anti-inflammatory, antifibrotic, and anti-apoptotic capacities (Cao et al., 2012). It can exert its antioxidant effects by directly scavenging ROS or by increasing the expression of different antioxidant enzymes such as SOD, GPx, and HO-1 or inhibiting the expression of Nox2 and Nox4; being the regulation of the Nrf2 pathway the core target of these antioxidant mechanisms (Xiao et al., 2020) (Figure 2).

##### 2.2.3.1 *In vitro* Cellular Studies

Sal B was found to inhibit TGF-β1-induced cell proliferation, differentiation, expression collagen type I, endogenous TGF-β1 production, and α-SMA expression in lung fibroblasts (Jiang et al., 2009; Zhang et al., 2011; Zhang et al., 2014b; Liu et al., 2016c), as well as EMT of alveolar epithelial cells (Liu et al., 2016c). A recent study has demonstrated that Sal B exerts an anti-inflammatory role by protecting endothelial cells from oxidative stress injury (Liu et al., 2018b). A further study has also demonstrated that Sal B inhibits LPS-induced inflammation *in vitro*, by down-regulating the protein expression of pro-inflammatory cytokine such as IL-1β and TNF- α (Jiang et al., 2020). Sal B treatment was also reported to reduce ROS production and inhibit myofibroblast transdifferentiation *via* the up-regulation of the Nrf2 pathway in human lung fibroblasts (Liu et al., 2018a).

##### 2.2.3.2 *In vivo* Animal Studies

Results from various bleomycin-induced pulmonary fibrosis *in vivo* studies showed that Sal B treatment ameliorates lung fibrosis, inhibits inflammatory cell infiltration and diminishes inflammatory cytokine production. It also reduces collagen accumulation and α-SMA expression, increases the expression of Nrf2 and protects endothelial cells against oxidative stress injury and inhibits endothelial cell apoptosis (Liu et al., 2015; Liu et al., 2016c; Liu et al., 2018b; Zhang et al., 2021). Sal B was also reported to protect against paraquat-induced pulmonary fibrosis by mediating Nrf2/Nox4 redox balance, as increased Nrf2 expression and reduced Nox4 one, and TGF-β1/Smad3 signalling (Liu et al., 2016a).

##### 2.2.3.3 Human Clinical Trials

As we have reviewed above, Sal B has been reported to reduce different fibrotic processes such as proliferation, TGF-β1 expression, EMT or myofibroblast transdifferentiation in *in vitro* models of fibrosis. *In vivo* studies have also demonstrated that Sal B protects from fibrosis and inflammation in bleomycin and paraquat models probably by upregulating Nrf2. As it is usual in this kind of compound, there is no clinical trial testing the effects of Sal B on its own.

Nevertheless, in 2017 it was started an open label clinical trial of Chinese herbal medicine for IPF (“The Effectiveness of an Empirical Chinese Medicine Formulation for Idiopathic Pulmonary Fibrosis: an Open Label Clinical Trial”), whose aim was to determine whether treatment with the herbal formula PROLUNG could improve IPF symptoms, respiratory function and the quality of life compared with pretreatment baseline. The primary outcome was the annual rate of change in FVC. The PROLUNG formula contained, among others, Radix *Salviae Miltiorrhizae,* the main bioactive compound of which is Sal B. Unfortunately, the trial was terminated in 2021 due to difficulties recruiting eligible patients (ClinicalTrials.gov Identifier: NCT03274544) Summarized in Table 4.

#### 2.2.4 Epigallocatechin Gallate

**FIGURE 3:**
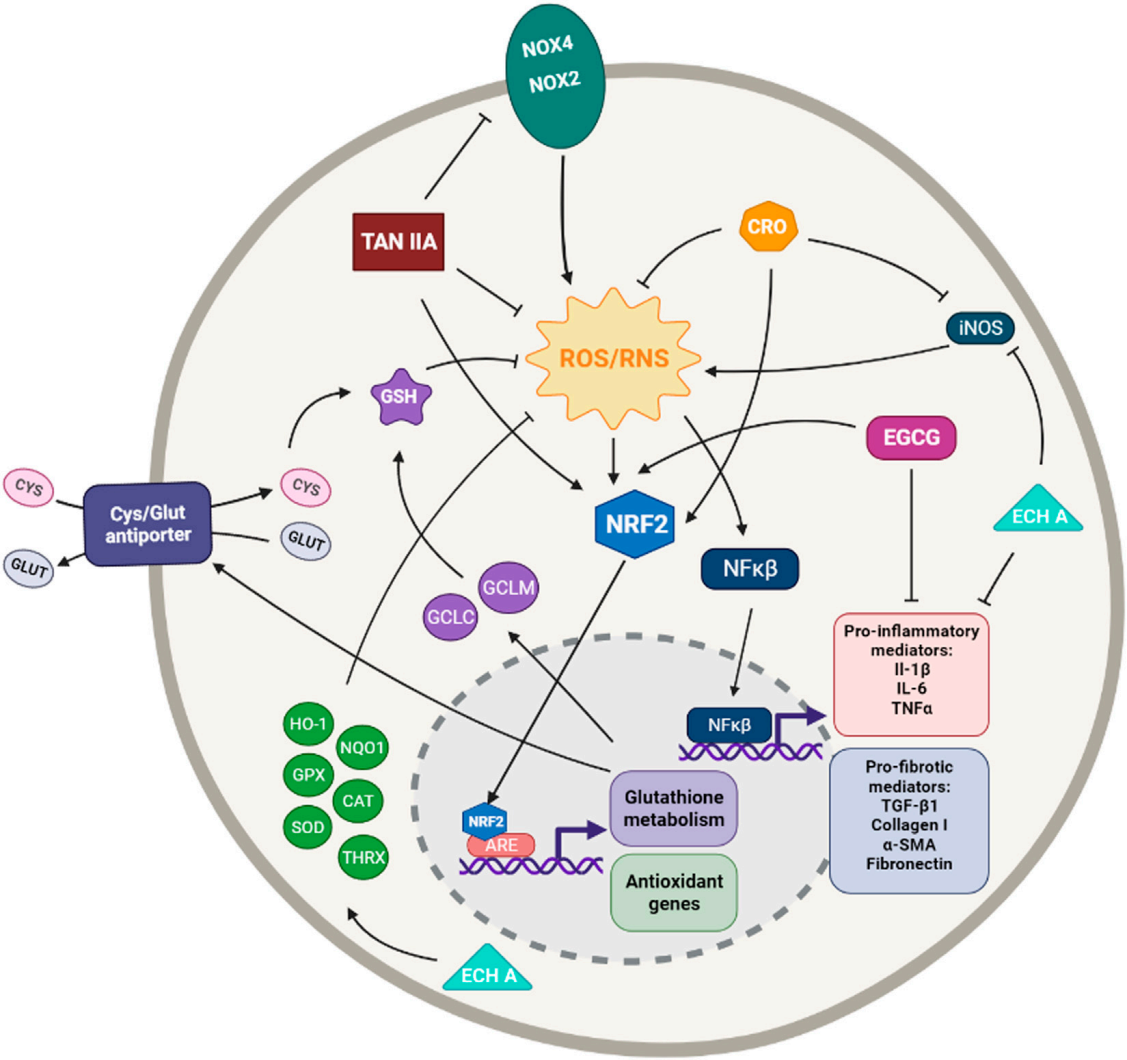
Simplified diagram of the principal molecular mechanisms of the antioxidant enhancers Epigallocatechin (EGCG), Tanshinone IIA (TAN IIA), Crocin (CRO) and Echinochrome A (ECH A). αSMA: alpha smooth muscle actin; ARE: antioxidant responsive element; CAT: catalase; CYS: cysteine; GCLC: glutamate cysteine ligase catalytic subunit; GCLM: glutamate cysteine ligase modifier subunit; GLUT: glutamate; GPX: glutathione peroxidase; GSH: glutathione; HO-1: heme oxygenase 1; IL-1β: interleuquina 1beta; IL-6: interleuquina 6; iNOS: inducible nitrogen oxide synthase; NFκβ: nuclear factor kappa beta; NOX1,2,4: NADPH oxidases; NQO1: NAD(P)H:quinone oxidoreductase 1; NRF2: nuclear factor erythroid 2-related factor 2; ROS/RNS: reactive oxygen species/reactive nitrogen species; TGF-β1: transforming growth factor beta 1; TNFα: tumoral necrosis factor alpha; SIRT: sirtuine 1; SOD: supeoxide dismutase Created with Biorender.com.

Epigallocatechin gallate (EGCG) is a polyphenol, the ester from epigallocatechin and gallic acid, and the major biological component of green tea, even though it can be also found in some other vegetables such as onions, hazelnuts and plums (Tsai et al., 2019). EGCG has a potent radical scavenging activity towards both superoxide and hydroxyl radicals, as well as, peroxyl radicals, nitric oxide, carbon-centered ROS and lipid oxidation products (Salah et al., 1995; Nanjo et al., 1999; Zhang et al., 2007a). Several studies have shown that EGCG acts not only as an antioxidant but also as an antiapoptotic, anti-inflammatory and antifibrotic agent (Nagai et al., 2002a; Sriram et al., 2009b; Oz, 2017; Minnelli et al., 2018). Another study suggests that EGCG can act as an antioxidant directly but also indirectly by increasing the activity of other antioxidants or enzymes (Negishi et al., 2004; Zhang et al., 2007a). This catechin may also participate in the regulation of mitochondrial metabolism (Shi et al., 2018b) (Figure 3).

##### 2.2.4.1 *In vitro* Cellular Studies

The protective role of EGCG was investigated *in vitro* inducing fibroblast cell lines with TGF-β1. Simultaneous treatment with EGCG inhibited the increased fibroblast proliferation, reduced hydroxyproline levels and decreased expression of MMP-2 and -9, *p*-Smad, α-SMA and type I collagen. Thus, EGCG inhibited fibroblast activation and collagen accumulation by inhibiting TGF-β1 signalling (Sriram et al., 2015). Another experimental study explored the effect of EGCG on gene expression in pulmonary fibroblasts from IPF patients. The gene expression changes observed were mainly involved in the biosynthesis and metabolism of cholesterol, suggesting that EGCG may exercise its effects through regulation of the cholesterol-associated genes (Tsai et al., 2019).

Despite there are not many *in vitro* studies that explore the therapeutic potential of EGCG in pulmonary fibrosis, there are several that investigate its effects in inflammatory processes. For instance, EGCG is shown to inhibit neutrophil elastase and elastase-mediated activation of MMP-9 (Sartor et al., 2002). Another *in vitro* study has also revealed that EGCG suppresses ROS activity and inhibits apoptosis and chemokine-induced chemotaxis in activated neutrophils (Donà et al., 2003). In addition, EGCG is demonstrated to reduce neutrophil transmigration through monolayers of endothelial cells (Hofbauer et al., 1999).

##### 2.2.4.2 *In vivo* Animal Studies

In 2003, Donà et al. (2003) demonstrated that both oral EGCG and green tea extract block neutrophil recruitment and neutrophil-mediated angiogenesis *in vivo* in an inflammatory angiogenesis mouse model induced by macrophage inflammatory protein-2 and LPS. They also showed that oral administration of green tea extract reduced inflammatory cell infiltration and the patchy fibrosis in pulmonary fibrosis and inflammation mouse model induced by intratracheal instillation of fluorescein isothiocyanate (FITC) (Donà et al., 2003).

Further studies demonstrated that EGCG administration alleviates the oxidative stress generated during bleomycin-induced pulmonary fibrosis in rat models. Furthermore, administration of this compound improved body weight and enzymic and non-enzymic antioxidants. It also decreased levels of ROS, lipid peroxidation, hydroxyproline, and the activity of myeloperoxidase. On the other hand, it increased cell counts. EGCG treatment also decreased the increased expression of nuclear factor-kB, TNF-α, IL-1b, MMP-2, and 9, TGF-β1, Smads, and α-SMA; restored the activities of antioxidant enzymes such as GST and NQO1; and induced Nrf2 (Sriram et al., 2008; Sriram et al., 2009a; Sriram et al., 2015).

A study from 2014 showed that EGCG treatment provides antioxidant, anti-inflammatory, and anti-proliferative effects that protect against irradiation-induced pulmonary fibrosis in rats. Treatment with EGCG reduced mortality rates and lung index scores; improved histological changes in the lung; reduced collagen depositions and MDA content; enhanced SOD activity; inhibited (myo) fibroblast proliferation; protected alveolar epithelial type II (AE2) cells; and regulated serum levels of TGF-β1, IL-6, IL-10, and TNF-α. Treatment with EGCG also activated Nrf2 and its downstream antioxidant enzymes HO-1 and NQO-1 (You et al., 2014).

##### 2.2.4.3 Human Clinical Trials

It has been demonstrated, as we have reviewed above, in *in vitro* studies that treatment with EGCG inhibits fibroblast proliferation, among others fibrotic processes, and, mainly, inflammatory processes. In several *in vivo* assays, it has been reported that EGCG provides antioxidant, anti-inflammatory and antifibrotic protection in different IPF models mainly via activation of the Nrf2 pathway.

Based on of these preclinical studies, in 2018, started an open label trial to test the effects of oral EGCG treatment on lung tissues and serum samples obtained from 20 patients with IPF and it is titled “Fibroblast Specific Inhibition of LOXL2 and TGFbeta1 Signaling in Patients With Pulmonary Fibrosis.” Half of the patients were given orally EGCG before they underwent biopsy, and the other half did not receive the treatment. EGCG treatment reversed profibrotic biomarkers in their diagnostic biopsies: type I collagen, snail family transcriptional repressor and phosphorylated SMAD3 levels were significantly lower in treated patients. This study is in an early phase; therefore, future results are expected in coming years (Chapman et al., 2020) (ClinicalTrials.gov Identifier: NCT03928847) Summarized in Table 4.

The same authors that conducted the open label study extended these findings to advanced pulmonary fibrosis using cultured precision-cut lung slices from explants of IPF patients undergoing transplantation. They discovered EGCG attenuate TGF-β1 signalling and new collagen accumulation and activated MMP-dependent collagen I turnover (Wei et al., 2021).

#### 2.2.5 Tanshinone IIA and Sodium Tanshinone IIA Sulfonate

Tanshinone IIA (TanIIA) is an active compound in *Salvia miltiorrhizae* Bunge*,* also known as Danshen, with numerous pharmacological activities, including antioxidant, anti-inflammatory, anticancer, and cardio-cerebrovascular protection activities (Cai et al., 2016). Due to the strong liposubility of TIIA, some researchers use its water-soluble derivative form, Sodium tanshinone IIA sulfonate (STS), which is reported to have superior bio-availability and similar pharmacological activity to TanIIA (Chen et al., 2016; Chen et al., 2017). It has also been reported that TanIIa exerts its cytoprotective effect through inhibition of ROS via activation of the Nrf2 pathway (Zhang and Wang, 2007) (Figure 3).

##### 2.2.5.1 *In vitro* Cellular Studies

TanIIA was reported to suppress TGF-β1-induced EMT and collagen I production in lung alveolar epithelial cells (Tang et al., 2015). Another study demonstrated in mouse embryonic fibroblasts that TanIIA inhibits myofibroblast activation through restoring redox homeostasis by activating Nrf2 and suppressing Nox4. Additionally, it was demonstrated TanIIA may activate Nrf2/GSH signalling to restrain myofibroblast proliferation by limiting glutamate availability to support cell growth (An et al., 2019). A more recent study has demonstrated that TanIIA inhibits silica-induced EMT and reduces oxidative stress *via* activation of the Nrf2 pathway in human alveolar epithelial cells and human bronchial epithelial cells (Feng et al., 2020b).

Similarly, STS was reported to ameliorate silica-induced cell proliferation and oxidative stress *via* activation of the Nrf2 and thioredoxin system in a coculture model of macrophages and pulmonary fibroblasts (Zhu et al., 2016). More recently, *in vitro* studies with pulmonary fibroblasts have reported that STS inhibits inflammation *via* downregulation of IL-1β and TNF-α. Additionally, STS was reported to inhibit TGF-β1-induced proliferation and α-SMA and collagen I overexpression in pulmonary fibroblasts (Jiang et al., 2020).

##### 2.2.5.2 *In vivo* Animal Studies

Wu et al. (2014) showed that TanIIA treatment attenuates bleomycin-induced pulmonary fibrosis and inflammation and decreases expression of TGF-β1 *via* modulating angiotensin-converting enzyme 2/angiotensin-(1–7) axis in rats. Later, other studies demonstrated that TanIIA mitigates bleomycin-induced pulmonary fibrosis, myofibroblast activation, collagen deposition, inflammatory cell infiltration and pro-inflammatory cytokine release in murine models (He et al., 2015; Tang et al., 2015; An et al., 2019). Additionally, it was also reported that TanIIA treatment reduces oxidative stress biomarker MDA levels and inhibits COX-2-associated oxidative reaction and iNOS-derived NO production (He et al., 2015). Furthermore, several studies have demonstrated that TanIIA attenuates silica-induced pulmonary fibrosis in rats via TGF-β1/Smad signalling suppression, Nox4 inhibition and Nrf2/ARE signalling activation. They also reported an Nrf2-mediated inhibition of EMT and a decrease in oxidative stress biomarkers (Feng et al., 2019; Feng et al., 2020a; Feng et al., 2020b).

In 1994, Wang, He and Zhang studied the effects of STS treatment against bleomycin-induced pulmonary fibrosis in rats. They revealed STS decreases levels of lipid peroxides and hydroxyproline, ameliorating fibrosis (Wang et al., 1994). More recent studies have demonstrated that STS reduces ROS and MDA production and collagen deposition, thereby attenuating silica-induced pulmonary fibrosis in rats, *via* activation of the Nrf2 and thioredoxin system (Zhu et al., 2016; Zhu et al., 2020).

##### 2.2.5.3 Human Clinical Trials

Despite the promising preclinical results that have demonstrated TanIIA activates the Nrf2 pathway and ameliorates different fibrotic processes in IPF models, there are no clinical trials on TanIIA. On the other hand, there are some clinical trials on STS regarding various diseases but none regarding pulmonary fibrosis.

#### 2.2.6 Resveratrol

**FIGURE 4:**
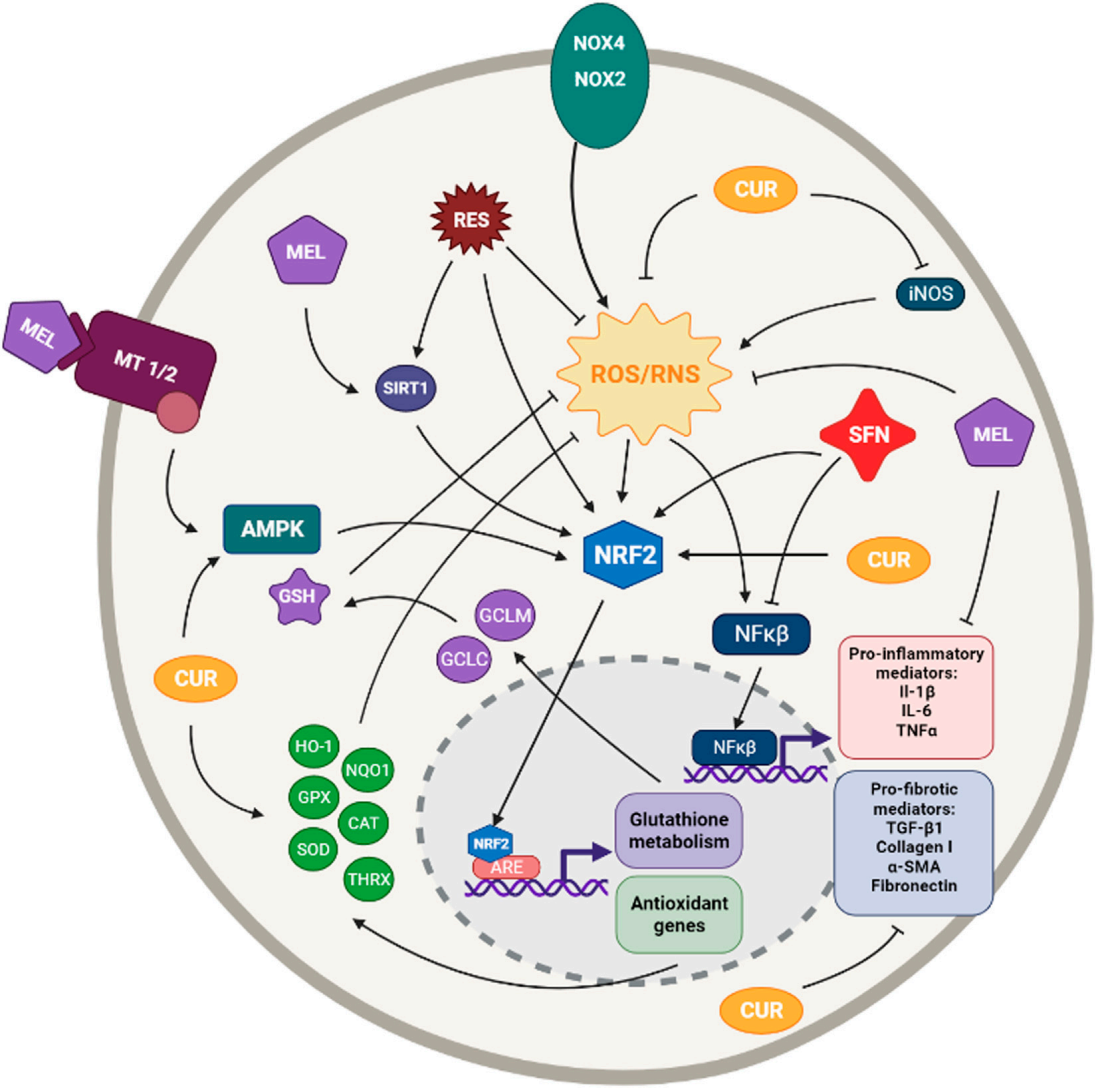
Simplified diagram of the principal molecular mechanisms of the antioxidant enhancers Resveratrol (RES), Sulforaphane (SFN), Melatonin (MEL) and Curcumin (CUR). αSMA: alpha smooth muscle actin; AMPK: AMP-activated protein kinase; ARE: antioxidant responsive element CAT: catalase; CYS: cysteine; GCLC: glutamate cysteine ligase catalytic subunit; GCLM: glutamate cysteine ligase modifier subunit; GLUT: glutamate; GPX: glutathione peroxidase; GSH: glutathione; HO-1: heme oxygenase 1; IL-1β: interleuquina 1beta; IL-6: interleuquina 6; iNOS: inducible nitrogen oxide synthase; NFκβ: nuclear factor kappa beta; NOX1,2,4: NADPH oxidases; NQO1: NAD(P)H:quinone oxidoreductase 1; NRF2: nuclear factor erythroid 2-related factor 2; ROS/RNS: reactive oxygen species/reactive nitrogen species; TGF-β1: transforming growth factor beta 1; TNFα: tumoral necrosis factor alpha; SIRT: sirtuine 1; SOD: supeoxide dismutase Created with Biorender.com.

Resveratrol (3,5,4-trihydroxystilbene) is a nonflavonoid polyphenolic compound found in multiple plant species, including grapes and peanuts. Resveratrol has been reported to exert a vast number of health benefits, such as antioxidant, anti-inflammatory, antifibrotic, antiviral, and anticancer activities, through many different mechanisms of action (Tomé-Carneiro et al., 2013; Ma and Li, 2020). Several studies have highlighted the therapeutic effects of resveratrol against lung diseases mainly by decreasing oxidant stress and inhibiting inflammatory responses (Conte et al., 2015; Ma and Li, 2020). One of the protective mechanisms of resveratrol par excellence is the activation of the Nrf2 pathway and, therefore, the induction of the expression of several antioxidants enzymes and the regulation of GSH homeostasis (Zhu et al., 2017) (Figure 4).

##### 2.2.6.1 *In vitro* Cellular Studies

Resveratrol was demonstrated to inhibit both fibroblast proliferation and differentiation into myofibroblast. In addition, different results demonstrated that resveratrol represses TGF-β1-induced collagen production, lung fibroblast proliferation (both normal and IPF lung fibroblasts) and attenuates α-SMA expression (Fagone et al., 2011). He et al. (2012) demonstrated that resveratrol suppresses oxidative stress and fibrogenic responses induced by paraquat (a fibrosis inducer herbicide) through activation of the Nrf2 pathway and, thus, inducing cytoprotective genes, such as HO-1 and NQO1. A posterior study showed resveratrol was able to repress and reverse myofibroblasts TGF-β- and/or CXCL12-mediated transformation (Mehrzadi et al., 2020). Resveratrol was also reported to inhibit the liberation of inflammatory cytokines such as IL-8 and IL-6 in lung epithelial cells (Gauliard et al., 2008).

##### 2.2.6.2 *In vivo* Animal Studies

Resveratrol has been shown to produce antifibrotic effects in murine models of various diseases such as renal, cardiac, and hepatic fibrosis (Conte et al., 2015). In the context of pulmonary fibrosis, resveratrol was demonstrated to ameliorate oxidative injury and fibrosis induced by bleomycin due to its antioxidant properties (Sener et al., 2007). Similarly, posteriors studies confirmed the promising potential of resveratrol on the treatment of fibrosis in the same bleomycin-induced pulmonary fibrosis rat model (Akgedik et al., 2012; Wang et al., 2018). Further studies demonstrated that treatment with resveratrol ameliorates LPS-induced EMT and pulmonary fibrosis through suppression of oxidative stress and TGF-β1/Smad signalling pathway (Zhang et al., 2015). Additionally, resveratrol has been reported to abolish bleomycin- and particulate matter-induced lung inflammation and fibrosis (Impellizzeri et al., 2015; Ding et al., 2019).

##### 2.2.6.3 Human Clinical Trials

It has been demonstrated resveratrol is able to inhibit several fibrotic processes through suppression of oxidative stress in *in vitro* and *in vivo* assays, as we have explained above. Nevertheless, it seems the obtained results have not provided sufficient preclinical evidence to consider resveratrol a good candidate for human trials. Indeed, none of the clinical trials on resveratrol registered in https://clinicaltrials.gov regards lung diseases.

#### 2.2.7 Sulforaphane

Sulforaphane (SFN) is an organosulfur compound, mainly found in cruciferous vegetables, with indirect antioxidant activity *via* the Nrf2-mediated induction of phase II detoxifying enzymes (Elbarbry and Elrody, 2011; Kim and Park, 2016) (Figure 4).

##### 2.2.7.1 *In vitro* Cellular Studies

SFN treatment was reported to decrease oxidants and to induce Nrf2 expression, antioxidants, and myofibroblast dedifferentiation in normal and IPF fibroblasts (Artaud-Macari et al., 2013). Various studies in human and rat alveolar epithelial cells have also demonstrated that activation of the Nrf2 antioxidant pathway by SFN protects against ROS production and TGF-β1-induced EMT (Zhou et al., 2016; Zhang et al., 2018d; Qu et al., 2019)**.** SFN has also been proved to attenuate TGF-β1-induced expression of fibrosis-related proteins, such as fibronectin, collagen I, collagen IV, and α-SMA in human alveolar epithelial cells and human fibroblasts (Kyung et al., 2018; Liu et al., 2021).

##### 2.2.7.2 *In vivo* Animal Studies

Several studies have reported that SFN has antifibrotic and antioxidant activity in various animal models (Elbarbry and Elrody, 2011). SFN treatment of bleomycin-induced pulmonary fibrosis attenuates fibrosis, apoptosis and lung oxidative stress by increasing the expression of antioxidant enzymes, NQO1, HO-1, SOD and catalase, *via* upregulation of Nrf2 gene expression (Yan et al., 2017). Another study reported that SFN decreases bleomycin-induced fibronectin expression, TGF-β1 expression, and the levels of collagen I in bleomycin-induced pulmonary fibrosis mouse model (Kyung et al., 2018).

##### 2.2.7.3 Human Clinical Trials

As we have mentioned above, several *in vitro* and *in vivo* studies using SFN have reported promising results in pulmonary fibrosis models *via* the activation of Nrf2, which is its main target. Nevertheless, there are no clinical trials on IPF. On the other hand, SFN has been used in several clinical trials to treat a wide variety of diseases such as cancer, neurological disorders, asthma, allergies and Chronic Obstructive Pulmonary Disease (COPD).

#### 2.2.8 Melatonin

Melatonin (N-acetyl-5-methoxytryptamine) is the main secretory product of the pineal gland and, along with its metabolites, is a potent antioxidant with lipophilic and hydrophilic characteristics (Reiter et al., 2016; Hosseinzadeh et al., 2018b). Melatonin can exert its antioxidant effects by either directly scavenging ROS and RNS or by indirectly up-regulating the expression and activities of endogenous antioxidants (Reiter et al., 2016). The antioxidant actions of melatonin are both receptor-dependent and independent (Reiter et al., 2007). In addition to regulating oxidative stress, melatonin modulates a variety of molecular pathways such as circadian biology, inflammation, proliferation, apoptosis and cellular injury (Wang, 2009; Jin et al., 2014; Vriend and Reiter, 2015; Hosseinzadeh et al., 2018a). Several studies have revealed that melatonin is able to induce the expression of GSH and various antioxidant enzymes such as catalase, SOD, and GPx, as well as to activate the Nrf2 signalling pathway (Swiderska-Kołacz et al., 2006; Santofimia-Castaño et al., 2015; Goc et al., 2017) (Figure 4).

##### 2.2.8.1 *In vitro* Cellular Studies

Various studies have reported that melatonin treatment upregulates Nrf2 expression, reduces ROS production and MDA levels, and prevents LPS- or TGF-β1-induced EMT in alveolar epithelial cells (Yu et al., 2016; Ding et al., 2020). Melatonin has also been reported to inhibit the expression of TGF-β1, collagen I and SMAD3 phosphorylation in pulmonary cells exposed to cigarette smoke and LPS (Shin et al., 2017). Different studies have also demonstrated that melatonin suppresses acrolein-induced IL-8 production in human pulmonary fibroblasts (Kim et al., 2012) and inhibits TGF-β1-induced fibrogenesis in mouse lung fibroblasts (Zhao et al., 2018). It also attenuates chromium-induced lung injury by reducing the production of oxidative stress and inflammatory mediators and by inhibiting cell apoptosis *via* activation of the Nrf2 signalling pathway in mouse lung epithelial cells (Han et al., 2019). Additionally, it has been reported that lipid-core nanocapsules of melatonin reduced oxidative DNA damage detected in alveolar epithelial cells treated with paraquat (Charão et al., 2015).

##### 2.2.8.2 *In vivo* Animal Studies

Several studies have investigated the effects of melatonin on bleomycin-induced pulmonary fibrosis in murine models and have found that melatonin prevents lung fibrosis development by suppressing oxidative stress and protein and lipid peroxidation (Arslan et al., 2002; Genovese et al., 2005; Yildirim et al., 2006; Karimfar et al., 2015; Zhao et al., 2018). Melatonin has also been reported to inhibit endoplasmic reticulum stress and EMT in bleomycin-induced pulmonary fibrosis in mice (Zhao et al., 2014). Furthermore, treatment with melatonin has been shown to attenuate chromium-induced lung injury *via* activating the Nrf2 pathway in rats (Han et al., 2019) and also to reduce leukocyte and macrophage inflammation and fibrosis in carbon tetrachloride-induced oxidative lung damage in rats (Taslidere et al., 2014).

##### 2.2.8.3 Human Clinical Trials

Despite the several *in vitro* and *in vivo* assays demonstrating that melatonin reduces different processes involved in fibrosis, such as apoptosis or fibrogenesis, there are no clinical trials regarding pulmonary fibrosis.

#### 2.2.9 Curcumin

Curcumin (diferuloylmethane) is a polyphenol compound, contained in the spice turmeric and isolated from the rhizome of the plant Curcuma longa. Curcumin has been found to have a multitude of pharmacological properties such as anticancer, antiviral, antiarthritic, anti-amyloid, antioxidant, and anti-inflammatory properties. The underlying mechanisms of these effects appear to involve the regulation of various molecular targets, including transcription factors, growth factors, inflammatory cytokines, protein kinases and other enzymes. Curcumin exerts its antioxidant activities directly, by scavenging superoxide anion and hydroxyl radicals, and indirectly by, among others, activating the Nrf2 pathway and inducing the expression of different antioxidant enzymes such as HO-1 (Lee et al., 2010b; Zhou et al., 2011; Lelli et al., 2017) (Figure 4).

##### 2.2.9.1 *In vitro* Cellular Studies

Several studies demonstrated that curcumin inhibits collagen I deposition, expression of α-SMA and vimentin, as well as proliferation and differentiation in TGF-β-induced human and mouse lung fibroblasts or IPF fibroblasts (Smith et al., 2010; Liu et al., 2016b; Chen et al., 2019; Saidi et al., 2019; Chun-Bin et al., 2020). Curcumin has also been reported to protect lung mesenchymal stem cells from H_2_O_2_ and to block the radiation-induced generation of ROS by upregulating the expression of Nrf2 and HO-1 (Lee et al., 2010b; Ke et al., 2020).

##### 2.2.9.2 *In vivo* Animal Studies

Numerous *in vivo* experiments have demonstrated the antifibrotic potential of curcumin. Treatment with curcumin has been reported to inhibit the release of inflammatory cytokines, the expression of fibronectin, vimentin and TGF-β1, the hydroxyproline content, the ECM accumulation, the collagen I and IV deposition, the production of TNF-α, superoxide, NO and MDA as well as to raise the expression of SOD and GPX in bleomycin-induced pulmonary fibrosis murine models (Punithavathi et al., 2000; Zhou et al., 2006a; Zhou et al., 2006b; Zhang et al., 2007b; Xu et al., 2007; Chen et al., 2008; Zhao et al., 2008; Smith et al., 2010; Durairaj et al., 2020).

Other pulmonary fibrosis models such as paraquat-, silica particles-, radiation-, aspiration material- and amiodarone-induced models have revealed that curcumin is able to reduce the levels of TGF-β1, TNF-α, IL-6 and collagen type I, and to decrease the generation of ROS, hydroxyproline content, MDA and MPO and iNOS activity, to increase the activity of SOD, GPx and HO-1 (Thresiamma et al., 1998; Punithavathi et al., 2003; Xu et al., 2007; Guzel et al., 2009; Jiang et al., 2009; Lee et al., 2010b; Cho et al., 2013; Li et al., 2015; Barsan et al., 2021).

##### 2.2.9.3 Human Clinical Trials

Curcumin has been reported to have antioxidant and antifibrotic potentials both *in vitro* and in different animal models, as we have seen before. Furthermore, it has been tested in various clinical trials for the treatment of different lung diseases, especially COPD, but none of the clinical trials regards pulmonary fibrosis.

#### 2.2.10 Pirfenidone

**FIGURE 5:**
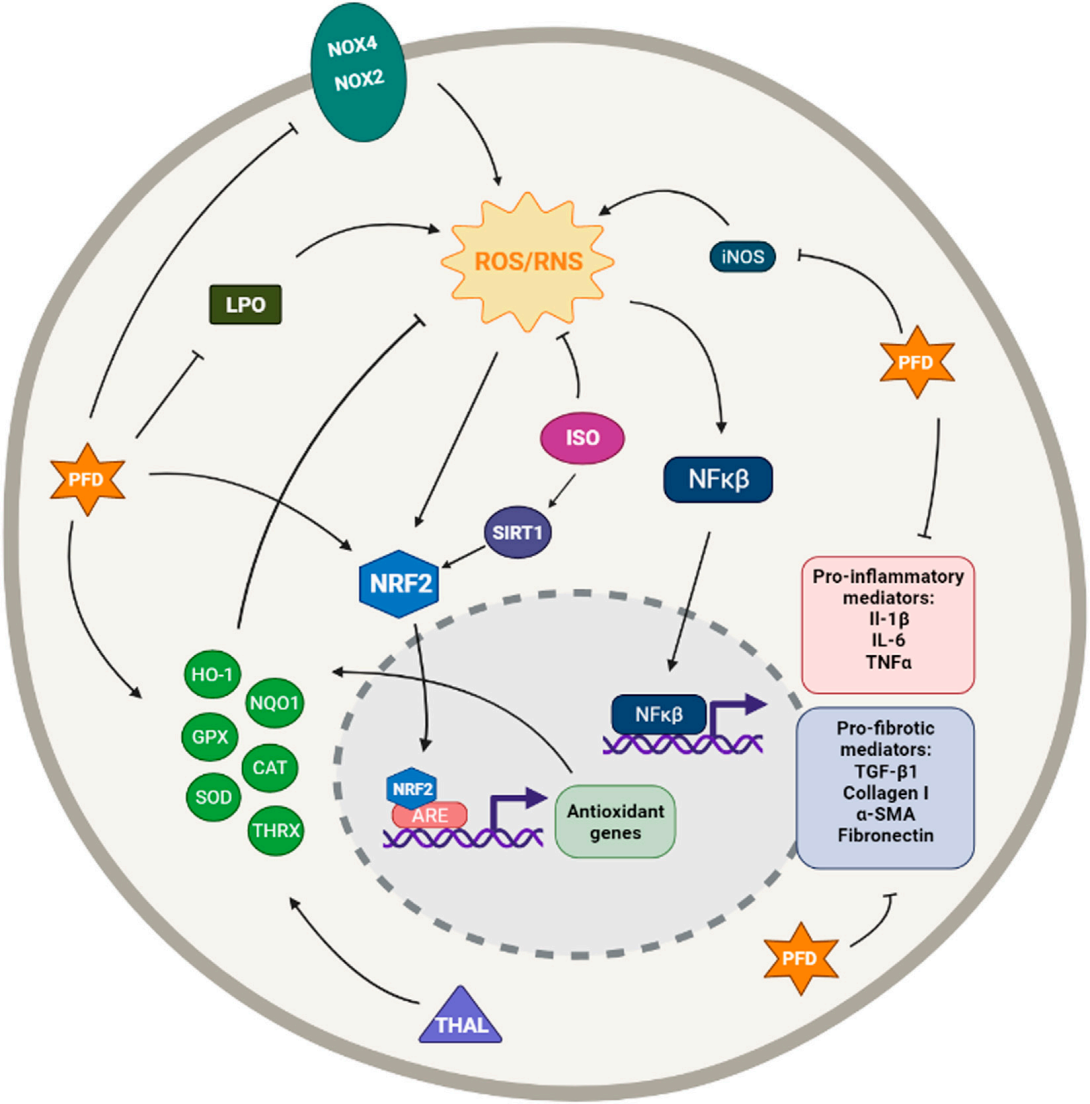
Simplified diagram of the principal molecular mechanisms of the antioxidant enhancers Pirfenidone (PFD), Thalidomide (THAL) and Isorhamnetin (ISO). αSMA: alpha smooth muscle actin; ARE: antioxidant responsive element; CAT: catalase; CYS: cysteine; GLUT: glutamate; GPX: glutathione peroxidase; HO-1: heme oxygenase 1; IL-1β: interleuquina 1beta; IL-6: interleuquina 6; iNOS: inducible nitrogen oxide synthase; LPO: lipoperoxidation; NFκβ: nuclear factor kappa beta; NOX1,2,4: NADPH oxidases; NQO1: NAD(P)H:quinone oxidoreductase 1; NRF2: nuclear factor erythroid 2-related factor 2; ROS/RNS: reactive oxygen species/reactive nitrogen species; TGF-β1: transforming growth factor beta 1; THRX: thioredoxin; TNFα: tumoral necrosis factor alpha; SIRT: sirtuine 1; SOD: supeoxide dismutase Created with Biorender.com.

Pirfenidone (PFD, 5-methyl-1-phenyl-2-[1H]-pyridone) is an oral broad-spectrum drug and is one of the two pharmacological treatments recommended for the treatment of IPF. Although no specific mode of action has been identified, PFD possesses antifibrotic, anti-inflammatory, and antioxidant properties (Poletti et al., 2014; Ruwanpura et al., 2020) (Figure 5).

##### 2.2.10.1 *In vitro* Cellular Studies

Regarding its antifibrotic potential, cell culture experiments on human lung fibroblasts and alveolar epithelial cells have revealed that PFD is able to inhibit fibroblast proliferation, myofibroblast differentiation, collagen synthesis, fibronectin production, deposition of ECM components, and α-SMA and HSP47 expression (Nakayama et al., 2008; Conte et al., 2014; Ma et al., 2018; Molina-Molina et al., 2018). Various studies have reported that PFD may inhibit the release of proinflammatory cytokines such as IL-6, IL-8, IL-1β and TNF-α and enhance the release of the anti-inflammatory cytokine IL-10, thereby exerting its anti-inflammatory effect (Nakazato et al., 2002; Oku et al., 2002; Spond et al., 2003; Liu et al., 2017a; Ruwanpura et al., 2020).

Regarding its antioxidant properties, PFD has been reported to reduce ROS production, as it is a scavenger of hydroxyl and superoxide anion free radicals (Giri et al., 1999). It has also been reported to inhibit NADPH-dependent lipid peroxidation in sheep liver microsomes (Misra and Rabideau, 2000). A more recent study has reported that PFD improves Nrf2, HO-1 and GPX1 expression and reduces collagen I and Il-6 levels in TGF-β1-induced mouse lung fibroblasts (Liu et al., 2017b). A further study has revealed that, contrary to sera from IPF naive patients, sera from PFD treated IPF patients failed to significantly induce both ROS generation and collagen synthesis in primary human pulmonary artery smooth muscle cells, demonstrating the antioxidant properties of PFD (Fois et al., 2018). PFD has also been reported to increase the expression of SOD1, catalase and Nrf2, as well as reduce the increased level of ROS in alveolar epithelial cells exposed to cigarette smoke extract (Ma et al., 2021).

##### 2.2.10.2 *In vivo* Animal Studies

Several studies have demonstrated the antifibrotic and anti-inflammatory effects of PFD in experimental animal models, especially in bleomycin-induced pulmonary fibrosis models. These studies revealed that PFD prevents the accumulation of hydroxyproline, collagen I and III, inflammatory cells and TGF-β1 in BALF, and/or lung tissue (Iyer et al., 1995; Iyer et al., 2000; Tian et al., 2006; Oku et al., 2008; Myllärniemi and Kaarteenaho, 2015).

In the 1990s, studies in hamster models of fibrosis indicated that PFD ameliorates bleomycin-induced lung fibrosis by suppressing oxidative stress mediators, such as MDA and MPO, and enhancing SOD activity (Iyer et al., 1995; Iyer et al., 1999). PFD has also been reported to attenuate bleomycin-induced pulmonary fibrosis in mice by improving the expression of Nrf2, HO-1 and GPx1 and reducing levels of ROS and MDA in serum, BALF and lung tissues (Liu et al., 2017b). In 2018, a study investigated the effect of PFD in paraquat-induced lung injury and fibrosis in mice and demonstrated that PFD ameliorates lung injury and fibrosis through inhibition of inflammation and oxidative stress, downregulation of profibrotic cytokines and enzymes for ROS production such as Nox1, Nox4, iNOS and through up-regulation of antioxidant enzymes such as SOD, catalase and GPx1 (Pourgholamhossein et al., 2018).

##### 2.2.10.3 Human Clinical Trials

The antioxidant and antifibrotic potential of PFD has been extensively demonstrated both *in vitro* and *in vivo* models of IPF, even though we have only highlighted the results regarding its antioxidant potential. The significant number of evidence made PFD a perfect candidate for human clinical trials.

Although there are multiple trials to test the efficacy of PFD in humans for IPF treatment, only four phase III clinical studies of PFD in IPF have been completed and reported (Liu et al., 2017a). Two open-label studies described for the first time the promising results of PFD for the treatment of IPF (Raghu et al., 1999; Nagai et al., 2002b). Later, a phase III trial was conducted in Japan and demonstrated that PFD preserves VC, the primary outcome, and improves progression-free survival time, the second outcome in mild IPF patients (Taniguchi et al., 2010).

Between 2006 and 2011 the CAPACITY (Clinical Studies Assessing Pirfenidone in idiopathic pulmonary fibrosis: Research of Efficacy and Safety Outcomes) phase III trial was conducted. This programme included two similar multinational trials (studies 004 and 006). The results of the two integrated trials showed a significant improvement in the lung function, the primary outcome, but also in progression-free survival time, in the change in FVC and in the associated death (Noble et al., 2011) (ClinicalTrials.gov Identifier: NCT00287716 and NCT00287729). Another phase III trial, ASCEND (Assessment of Pirfenidone to Confirm Efficacy and Safety in Idiopathic Pulmonary Fibrosis) study was conducted between 2011 and 2014. This trial confirmed that PFD reduces disease progression, as improved lung function, exercise tolerance, and progression-free survival (King et al., 2014) (ClinicalTrials.gov Identifier: NCT01366209) Summarized in Table 4.

#### 2.2.11 Thalidomide

Thalidomide (Thal, α-N-phthalimido glutarimide) is a glutamic acid derivative that was initially prescribed as a sedative and antiemetic drug but it was removed from the market for its teratogenic effects (Franks et al., 2004; Sharma et al., 2007). Thal possesses various pharmacological properties, including anti-inflammatory, antifibrotic and anti-angiogenic (Chong et al., 2006; Mondello et al., 2009; Zhang et al., 2018a). Although the mechanism of action that underlies these properties is still unclear, Thal may exert its biological activities by modulating inflammatory cytokines, growth factors and nitric oxide (Sampaio et al., 1991; Franks et al., 2004; Amirshahrokhi, 2013) (Figure 5).

##### 2.2.11.1 *In vitro* Cellular Studies

Thal was reported to reduce IL-18, IL-8 and TNF-α release from LPS-induced alveolar macrophages (Tavares et al., 1997; Ye et al., 2006). Additionally, several studies have demonstrated that Thal reduces the production of IL-6, TGF-β1, collagen type I and IV, α-SMA, vimentin, MMP-2 and -9, fibronectin and CTGF as well as to inhibit transdifferentiation, EMT and oxidative stress in TGF-β1-induced human and mouse lung fibroblasts (Tabata et al., 2007; Choe et al., 2010; Amirshahrokhi, 2013; Dong et al., 2017; Wu et al., 2020).

##### 2.2.11.2 *In vivo* Animal Studies

Various investigations have revealed that Thal prevents bleomycin- or paraquat-induced pulmonary fibrosis in murine models by downregulating the expression of IL-6, IL-8, IL-1β, TNF- α, TGF-β, collagen, hydroxyproline, VEGF, *p*-JNK and α-SMA (Tabata et al., 2007; Choe et al., 2010; Amirshahrokhi, 2013; Liu et al., 2014b; Dong et al., 2017). In addition, Thal has been reported to reduce MPO, NO, MDA and ROS and to enhance the activity of SOD and thioredoxin reductase in bleomycin- or paraquat-induced pulmonary fibrosis murine models (Amirshahrokhi, 2013; Dong et al., 2017).

##### 2.2.11.3 Human Clinical Trials

Thal is currently used in the treatment of multiple myeloma, but its use in other diseases is restricted due to its teratogenic effects (Cavallo et al., 2007). Nevertheless, since various preclinical studies demonstrated IPF is able to reduce different fibrotic processes, in 2005 it was started an open label phase II trial. This clinical trial aimed to determine whether Thal can stop the progression of fibrosis in IPF patients (“Treatment of Idiopathic Pulmonary Fibrosis With Thalidomide”). It was conducted between 2005 and 2010 and the primary outcome was to determine the safety, feasibility and efficacy of Thal. However, there are no results available about this study, probably because these are not promising results (ClinicalTrials.gov Identifier: NCT00162760).

On the other hand, there is a phase III trial testing the efficacy of Thal in suppressing the chronic cough of IPF (“Treatment of Chronic Cough in Idiopathic Pulmonary Fibrosis With Thalidomide”). The primary endpoint was the cough-specific quality of life measured by the Cough Quality of Life Questionnaire (CQLQ) and results showed that Thal improved cough and respiratory quality of life in IPF patients (Horton et al., 2012) (ClinicalTrials.gov Identifier: NCT00600028) Summarized in Table 4.

#### 2.2.12 Other Antioxidant Molecules

The antioxidant compounds reviewed above are the most studies ones of a large list of molecules able to enhance the antioxidant defence system against the fibrotic process. However, some antioxidant compounds have promising therapeutic results.

For instance, crocin, a natural antioxidant molecule, remarkably decreases TNF-α, MDA, TGF-β1 and NO levels, up-regulates Nrf2 and HO-1 and attenuates fibrosis in the lungs of bleomycin-exposed rats (Zaghloul et al., 2019; Mehrabani et al., 2020) (Figure 3). Another recently studied antioxidant is isorhamnetin, a flavonol aglycone isolated from the traditional Chinese medicine *Hippophae rhamnoides L*. (Shi et al., 2018a). Isorhamnetin has been demonstrated to inhibit bleomycin-induced collagen deposition, reduce type I collagen and α-SMA expression, and alleviate EMT and endoplasmic reticulum stress *in vivo* and *in vitro* (Zheng et al., 2019; Luo et al., 2019), as well as to decrease the expression of inflammatory cytokines (Ren et al., 2021; Chi et al., 2016) (Figure 5). Apart from these, another antioxidant molecule is echinochrome A, a quinoid pigment of marine invertebrates that exhibits potent antioxidant properties (Lebedev et al., 2005). It has been reported to have a direct cytoprotective effect under conditions of oxidative stress in pulmonary fibroblasts (Sazonova et al., 2020) and to reduce the severity of bleomycin-induced oxidative stress in the lungs of murine models (Lebed’ko et al., 2015) (Figure 3).

These are just a few examples of the studied antioxidant compounds but, indeed, the number of antioxidant components able to decrease fibrotic process through suppression of oxidative stress is countless and growing.

### 2.3 Superoxide Dismutase Mimetics

As we have mentioned above, SOD enzymes play a critical role in the antioxidant defence of the respiratory system. Thus, several of studies have investigated whether SOD enzymes administration can protect against oxidative stress and ameliorate some lung diseases. Treatment with SODs, encapsulated SODs, liposomal SOD preparations, and recombinant manganese superoxide dismutase, MnSOD, resulted to offer antioxidant protection in fibrosis models; however, these compounds developed some immunogenic complications (Kinnula and Crapo, 2003). Thereby, further studies have opted for synthetic small-molecular-weight SOD mimetics. These molecules include salen compounds, macrocyclics and metalloporphyrins and have been found to have antioxidant and anti-inflammatory properties (Salvemini et al., 2002).

**FIGURE 6:**
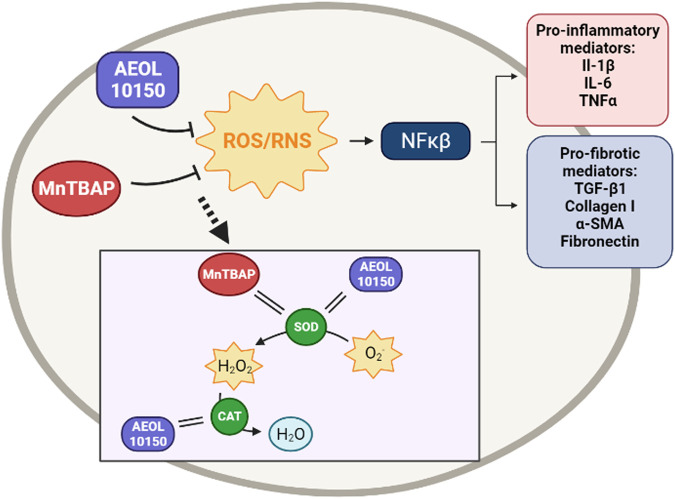
Simplified diagram of the principal molecular mechanisms of the SOD mimetics AEL10150 and MnTBAP. αSMA: alpha smooth muscle actin; CAT: catalase; IL-1β: interleuquina 1beta; IL-6: interleuquina 6; NFκβ: nuclear factor kappa beta; ROS/RNS: reactive oxygen species/reactive nitrogen species; TGF-β1: transforming growth factor beta 1; TNFα: tumoral necrosis factor alpha; SOD: supeoxide dismutase Created with Biorender.com.

AEOL 10150 is a broad-spectrum metalloporphyrin SOD mimic and several studies have shown that this agent protects lung tissues from radiation-induced injury in murine and primate models. It also reduces macrophages accumulation, oxidative stress, and collagen deposition (Rabbani et al., 2007; Garofalo et al., 2014; MacVittie et al., 2017; Zhang et al., 2018b). The metalloporphyrin AEOL 10113 has also been reported to reduce TGF-β levels and collagen deposition and to have a protective effect from radiation-induced lung injury (Vujaskovic et al., 2002). MnTBAP is another metalloporphyrin SOD mimic that has been demonstrated to attenuate bleomycin-induced pulmonary fibrosis in *in vitro* and *in vivo* models (Oury et al., 2001; Venkatadri et al., 2017). These compounds have; however, not yet been tested in human lung fibrosis (Figure 6).

## 3 Pitfalls of Antioxidant Therapies in Idiopathic Pulmonary Fibrosis

In this review, we have classified the antioxidant drugs into three classes: NOX inhibitors, antioxidants enhancers and ROS scavengers, and SOD mimetics. We have described the *in vitro* and *in vivo* results obtained with each drug in the treatment of pulmonary fibrosis. As well as a description of existing clinical trials, if any.

Although all these compounds may seem promising drugs for the treatment of fibrosis, most of them have critical drawbacks, as we will describe below.

Regarding the NOX inhibitors molecules, the classical molecules DPI and Vas2870 have been proven to be useful drugs in preclinical assays to elucidate the role of oxidative stress, specifically NOx molecules, in the development and progression of pulmonary fibrosis and could be helpful in the development of new drugs. However, both compounds have resulted not been suitable options for the treatment of IPF due to their lack of specificity, toxicity, and off-target effects, as they may have unpredictable effects in humans.

In contrast to DPI and Vas2870, the GKTs designed inhibitors are specific for Nox4/Nox1. As we have mentioned, the isoforms Nox1 and Nox4 are implicated in the pathogenesis of IPF. Nox4, in particular, is overexpressed in most of the cells implicated in the pathophysiology of IPF, such as (myo)fibroblasts and epithelial cells, and it contributes to the alteration of different processes such as migration, senescence, differentiation and EMT among others. It is, therefore, logical to think that Nox4 and Nox1 are promising drug targets for IPF. The specificity of these small molecules helps in avoiding off-target effects and in the determination of the effective dose, which is usually lower than in the case of broad-spectrum drugs. Preclinical assays both *in vitro* and *in vivo* have provided excellent results, or so the company has reported. Indeed, the GKT37831 compound has completed a phase I clinical trial with enough good results and a phase II clinical trial is ongoing. Thus, this small molecule is probably the most promising option for the treatment of IPF.

Apocynin had been considered as a NOX inhibitor for a long time; however, it has been recently demonstrated that it is a ROS scavenger. Therefore, like many other natural ROS scavengers, such as flavonoids, it has demonstrated beneficial effects in *in vitro* and *in vivo* models, but there is a lack of evidence for its efficacy in humans. The last NOX inhibitor mentioned in this review is metformin. In this case, *in vitro* and *in vivo* assays have shown that metformin is able to attenuate or reverse lung fibrosis processes; however, retrospective human clinical studies have reported that metformin is ineffective for the treatment of pulmonary fibrosis in diabetics patients with IPF.

Antioxidant enhancers and ROS scavengers are usually more tolerated than NOX inhibitors, but, on the other hand, their efficacy is also lower.

Among all the antioxidant enhancers, NAC is probably one of the most studied for the treatment of pulmonary fibrosis. Its promising results both *in vitro* and *in vivo* suggested that it could be an appropriate option for treating the oxidative stress occurring in IPF. Indeed, it has been reported to be effective in the treatment of other diseases such as Chronic Obstructive Pulmonary Disease (COPD) or bronchitis. However, the successive clinical trials have not reported successful results. On the other hand, the PRECISIONS trial has opened the door to a much more personalised therapy, based on the genetic characteristics of each patient and, probably, it is the key for the treatment of such diseases as IPF, which is characterised by the existence of several phenotypes. Additionally, another recently proposed study aims to test the effectiveness of NAC administered by inhalation. The change in the administration could improve its effectiveness as the drug would act directly in the lungs, where the damage is occurring. However, given all the results, treatment with NAC alone would be very unlikely to be effective in curbing fibrosis.

Many of the antioxidant compounds studied in this review are natural flavonoid or polyphenols compounds such as Quercetin, Sal B, EGCG, TanIIA and STS, resveratrol and curcumin. These compounds have shown a strong antioxidant and, in many cases, anti-inflammatory power in *in vitro* and *in vivo* preclinical fibrotic models however their clinical utility as antifibrotic agents is more than questionable. Natural compounds like these are usually very well tolerated with no toxic effects, but, on the other hand, they are less effective than other chemical derived drugs. Furthermore, they have no specific mode of action: they can have a multitude of targets or be ROS scavengers by themselves. The lower efficacy together with the lack of specificity makes it difficult to establish an effective dose to reach an acceptable clinical efficacy. Therefore, these drugs are more indicated for prevention than for treatment of diseases such as IPF and they could be useful in combination with other antifibrotic drugs.

Despite de several *in vitro* and *in vivo* assays demonstrating the benefit of using these kinds of antioxidants to treat diseases such as IPF, the reality is that the use of antioxidants in clinical trials has shown to be predominately ineffective (Steinhubl, 2008). The lack of benefit in clinical trials could have different explanations. The easiest explanation is that the chosen compound is not adequate for the disease. Another potential explanation may be that although the compound is adequate, it is tested at inappropriate doses or for inadequate durations (Steinhubl, 2008; M Davies and G Holt, 2018). In most cases, antioxidant compounds need unrealistic doses to have a physiological effect, indeed, it has been reported that they would be present at greater than 10^13^ molecules per cell (M Davies and G Holt, 2018). This could be not only because their efficacy is often lower than other drugs, but also because their consumption by ROS is more rapid. Furthermore, it seems illogical to use antioxidant therapies over just a few years to reverse the results of several decades of oxidative stress.

In this review, we also mentioned other three natural compounds, crocin, isorhamnetin and echinocrome A. These three compounds have shown a high antioxidant potential and, since they are natural, are unlikely to generate toxic effects. However, *in vitro* and *in vivo* assays with these compounds are scarce, thus, further evidence of their efficacy in fibrotic processes would be needed before they could be considered for clinical trials.

We consider that some of the antioxidants reviewed here deserve special mention, as is the case of SFN. This compound is a natural derivative, but unlike other drugs of this type, has more specific molecular targets. This is an advantage in establishing a dose that achieves sufficient efficacy in humans. One of its most important targets is Nrf2, a key factor in antioxidant defences and highly implicated in diseases related to oxidative stress, for instance, its expression is decreased in some IPF effector cells. Therefore, SFN, since it activates Nrf2, would be a possible candidate for human clinical trials regarding IPF, although more preclinical *in vitro* and *in vivo* trials may be needed beforehand.

Another drug that we would like to discuss separately is melatonin, as its origin is different from the rest. Melatonin is a hormone that has been shown to have strong antifibrotic effects due to its antioxidant and anti-inflammatory potential. As it is a hormone produced by the body itself, its use as a drug must be well controlled. Thus, concentration and dose (single or chronic) may be carefully studied. Chronic treatment with melatonin may have the opposite of the desired effects, so it may not be a good option for the treatment of fibrosis, since is a chronic disease. Furthermore, melatonin is heavily involved in circadian rhythms and altering its levels in the body can have detrimental effects. On the other hand, its use could be useful at specific times during the treatment of fibrosis.

In this review, we have also mentioned the PFD, one of the antifibrotic drugs par excellence and one of the two currently approved drugs for the treatment of IPF. As we have reviewed here, several *in vitro* and *in vivo* studies have shown that PFD has potent antioxidant effects. However, probably, a large part of these effects is due to its main antifibrotic effect. PFD has several mechanisms of action, many of which are still unknown. Due to the difficulty in determining its specific mechanisms of action, it cannot be said PFD to be a pure antioxidant drug or to have a main antioxidant power.

Another of the drugs already used in clinical practice that we have discussed in this review is Thal. In this case, Thal, in addition to its teratogenicity, which severely limits its use, has been reported ineffective in treating IPF in humans in different clinical trials. Although preclinical studies may suggest that it has anti-fibrotic effects, the reality is that the use of thalidomide for the treatment of IPF is out of the question. Thal is being tested to treat the chronic cough associated with IPF, however, four clinical studies are currently underway investigating P2X3-receptor antagonists as potential antitussives, the results are promising and they are safer than Thal (Dicpinigaitis et al., 2020). Therefore, it would also make no sense to use Thal to treat chronic cough when these antagonists are safer and more effective.

Finally, we have discussed some recently designed SOD mimetics, such as AEOL10150, AEOL 10113 and MnTBAP. These compounds, in contrast to most of the seen above, have a more specific mechanism of action, making it easier to determine the most effective concentration. Furthermore, the use of designed compounds that mimic a biological molecule avoid the complications arising from the use of the biological molecule itself, that, in this case, would be, primarily, the SOD molecule. These compounds, together with the synthetic NOX-inhibitors GKTs, are possibly the ones with the most promising future. However, more research is needed on these compounds before they can be considered suitable for clinical trials.

## 4 Concluding Remarks

Idiopathic pulmonary fibrosis is the major idiopathic interstitial pneumonia with no cause identified and it is characterized by cellular proliferation, interstitial inflammation, and a chronic and progressive fibrosis. It usually does not respond to traditional therapies such as anti-inflammatory and immunomodulatory treatments. Several studies have suggested that increased oxidative stress may play a central role in the development and progression of IPF; indeed, cellular participants in lung fibrosis show, in most cases, an imbalance between oxidants and antioxidants. In addition, the massive number of oxidative stress biomarkers described in patients with IPF have evidenced the involvement of ROS in the pathogenesis of the disease. Regarding this, several ROS scavengers and modulators of the antioxidant machinery have been tested as potential therapeutics for lung fibrosis in preclinical *in vitro* and *in vivo* models and in different clinical trials. In this review, we have highlighted the role of oxidative stress in the pathology of lung fibrosis and its underlying mechanisms. However, the principal aim of this review is to analyze the mechanism of action, the efficacy, and the current status of different drugs able to protect against oxidative stress and act as antifibrotic therapy in IPF. Several more antioxidant molecules can be considered as potential therapeutics for lung diseases but have not been included in this review due to the lack of enough experimental evidence in lung fibrosis; however, they can be consulted in different reviews (Kinnula et al., 2005; Day, 2008; Kato and Hecker, 2020; Wang et al., 2021).

Despite the evidence of the significance of oxidative stress in the progression and development of IPF, as well as the anti-fibrotic effect that many antioxidants have been reported to have, it is unlikely that antioxidant monotherapy will be able to reverse or halt IPF progression, as its efficacy is partial. However, the use of antioxidant drugs may serve to improve some aspects of the disease and may be useful in combination with anti-fibrotic drugs. This situation is not unique to IPF but also occurs with other complex diseases such as cancer (Hayes et al., 2020), where antioxidant drug treatments have been used also mainly for prevention or supplementation (Ozben, 2015; Athreya and Xavier, 2017).

Therefore, antioxidant therapies in IPF should probably be focused on complementing other more efficient antifibrotic therapies.

## Glossary

6MWD: 6-min walk distance
3-NT: 3-nitrotyrosine
AEC: alveolar epithelial cells
ARE: antioxidant response element
BALF: bronchoalveolar lavage fluid
b.i.d: bis in die, twice a day
COPD: chronic obstructive pulmonary disease
COX-2: cyclooxygenase 2
CQLQ: Cough Quality of Life Questionnaire
CRP -score: clinical, radiologic and physiologic score
DLCO: diffusing capacity of the lungs for carbon monoxide
DPI: Diphenyleneiodonium
EBC: expired breath condensate
ECM: extracellular matrix
EC-SOC: extracellular SOD
EGCG: Epigallocatechin gallate
ELF: epithelial lining fluid
EMT: epithelial-to-mesenchymal transition
eNOS: endothelial NOS
ERK: extracellular signal-regulated kinase
FEV: forced expiratory volume
FITC: fluorescein isothiocyanate
FVC: forced vital capacity
GCL: glutamate cysteine ligases
GCLC: glutamate cysteine ligase catalytic subunit
GCLM: glutamate cysteine ligase modifier subunit
GPx: glutathione peroxidase
GSH: glutathione
GSSG: oxidized glutathione
GST: glutathione S-transferase
HO-1: heme oxygenase I
HSP: heat shock protein
IL: interleuquin
iNOS: inducible NOS
IPF: idiopathic pulmonary fibrosis
JNK: c-Jun N-terminal kinase
KEAP1: Kelch like-ECH-associated protein 1
LPS: lipopolysaccharide
MDA: malondialdehyde
MMP: matrix metalloproteinase
MPO: myeloperoxidase
MUC: mucin
NAC: n-acetyl cysteine
NFκβ: nuclear factor kappa beta
nNOS: neural NOS
NO: nitric oxide
NOS: nitric oxide synthase
NOX: NADPH oxidase
NQO1: NADPH oxidoreductase I
NRF2: nuclear factor erythroid 2-related factor 2
PFD: pirfenidone
PFS: progression-free survival
PrxII: peroxiredoxin II
Quer: quercetin
RNS: reactive nitrogen species
ROS: reactive oxygen species
Sal B: Salvianolic acid B
SF-36: 36-Item Short Form Survey
SFN: sulforaphane
SGRQ: St. George’s Respiratory Questionnaire
SOD: superoxide dismutase
SpO2: oxygen saturation by pulse oximetry
SRX1: sulfiredoxin-1
STS: sodium anshinone IIA sulfate
TAC: total antioxidant capacity
TanIIA: tanshinone IIA
TBARS: thiobarbituric acid reactive substances
TEAC: trolox equivalent antioxidant capacity
TGF-β: transforming growth factor beta
tGSH: total glutathione
Thal: thalidomide
t.i.d: ter in die, three times a day
TLC: total lung capacity
TNF-α: tumoral necrosis factor Alpha
TOLLIP: toll-interacting protein
TRX: thioredoxin
VAS: visual analog scale of cough
VC: vital capacity
VSMCs: vascular smooth muscle cells
WHOQOL-BREF: World Health Organization Quality of Life abbreviated version
α-SMA: smooth muscle actine alpha
